# Targeting Cathepsin B with *p*-coumaric acid rescues WWP1-dependent proteasomal degradation of NLRP3 in hyperuricemic nephropathy

**DOI:** 10.1186/s10020-026-01544-y

**Published:** 2026-06-26

**Authors:** Fengqin Li, Yanzhe Wang, Xia Liu, Yue Wu, Naijun Miao, Jialing Wang, Xinyue Chen, Tong Wu, Qiao Fu, Chuchu Zeng, Nan Zhang, Xinmiao Xie, Xiaoxia Wang

**Affiliations:** 1 Department of Nephrology, Shanghai Tongren Hospital, Shanghai Jiao Tong University School of Medicine, 1111 XianXia Road, Shanghai, 200336 China; 2 National Clinical Research Center of Kidney Diseases, Jinling Hospital, Nanjing University School of Medicine, Nanjing, China; 3 Bengbu Medical University, Bengbu, 233000 Anhui China

**Keywords:** *p*-Coumaric acid, Hyperuricemic nephropathy, Cathepsin B, WWP1, Ubiquitination, NLRP3 inflammasome, Pyroptosis, Renal fibrosis

## Abstract

**Background:**

Hyperuricemic nephropathy (HN) is a prevalent metabolic disorder characterized by tubulointerstitial inflammation and fibrosis, driven primarily by aberrant NLR family pyrin domain containing 3 (NLRP3) inflammasome activation. However, effective therapies targeting this pathogenic axis remain elusive. We aimed to identify a pharmacological intervention for HN and elucidate its mechanisms.

**Methods:**

A targeted phenotypic screen of natural phenolic compounds was conducted against uric acid (UA)-induced inflammasome responses. Multi-omics profiling, molecular docking, and multi-dimensional biophysical assays—including surface plasmon resonance, cellular thermal shift assays, and circular dichroism—and site-directed mutagenesis were utilized to identify and characterize the direct molecular target. The underlying post-translational regulatory mechanism was investigated through co-immunoprecipitation and ubiquitination assays. In vivo efficacy and short-term tolerability were evaluated in a rat model of HN. Clinical relevance was assessed via renal biopsies and clinical sample analysis.

**Results:**

We identified *p*-coumaric acid (*p*-CA) as a potent pharmacological candidate. Mechanistically, *p*-CA engages the Cathepsin B (CTSB) active-site/occluding-loop region, involving residues Cys26 and His110, and suppresses CTSB enzymatic activity. Notably, we uncovered a post-lysosomal regulatory model in which UA-induced lysosomal CTSB leakage is associated with a CTSB activity-associated reduction in WW domain-containing E3 ubiquitin protein ligase 1 (WWP1) protein abundance/stability, thereby impairing WWP1-dependent K48-linked polyubiquitination and proteasomal clearance of NLRP3 and promoting inflammasome activation. *p*-CA-mediated CTSB inhibition preserves WWP1 protein abundance, restores NLRP3 proteasomal clearance, and attenuates downstream pyroptosis, inflammation, and fibrogenesis. CTSB knockdown attenuated the additional protective effect of *p*-CA, while pharmacological CTSB inhibition phenocopied *p*-CA, supporting CTSB pathway dependence. Furthermore, in vivo evaluations demonstrated that *p*-CA exerts urate-independent renoprotection without overt short-term systemic toxicities associated with synthetic CTSB inhibitors. Clinically, aberrant CTSB and NLRP3 upregulation correlated with impaired renal function in patients from the hyperuricemia-associated CKD cohort.

**Conclusions:**

Our findings support the CTSB-WWP1-NLRP3 regulatory axis as a previously unrecognized pathogenic mechanism of HN, revealing how lysosomal damage drives inflammasome hyperactivation by disrupting ubiquitin-dependent clearance. The precise molecular mechanism linking CTSB activity to WWP1 turnover remains to be fully defined. Targeting CTSB with *p*-CA provides a promising prophylactic or co-treatment candidate for HN warranting further translational evaluation.

**Graphical Abstract:**

**Figure:**
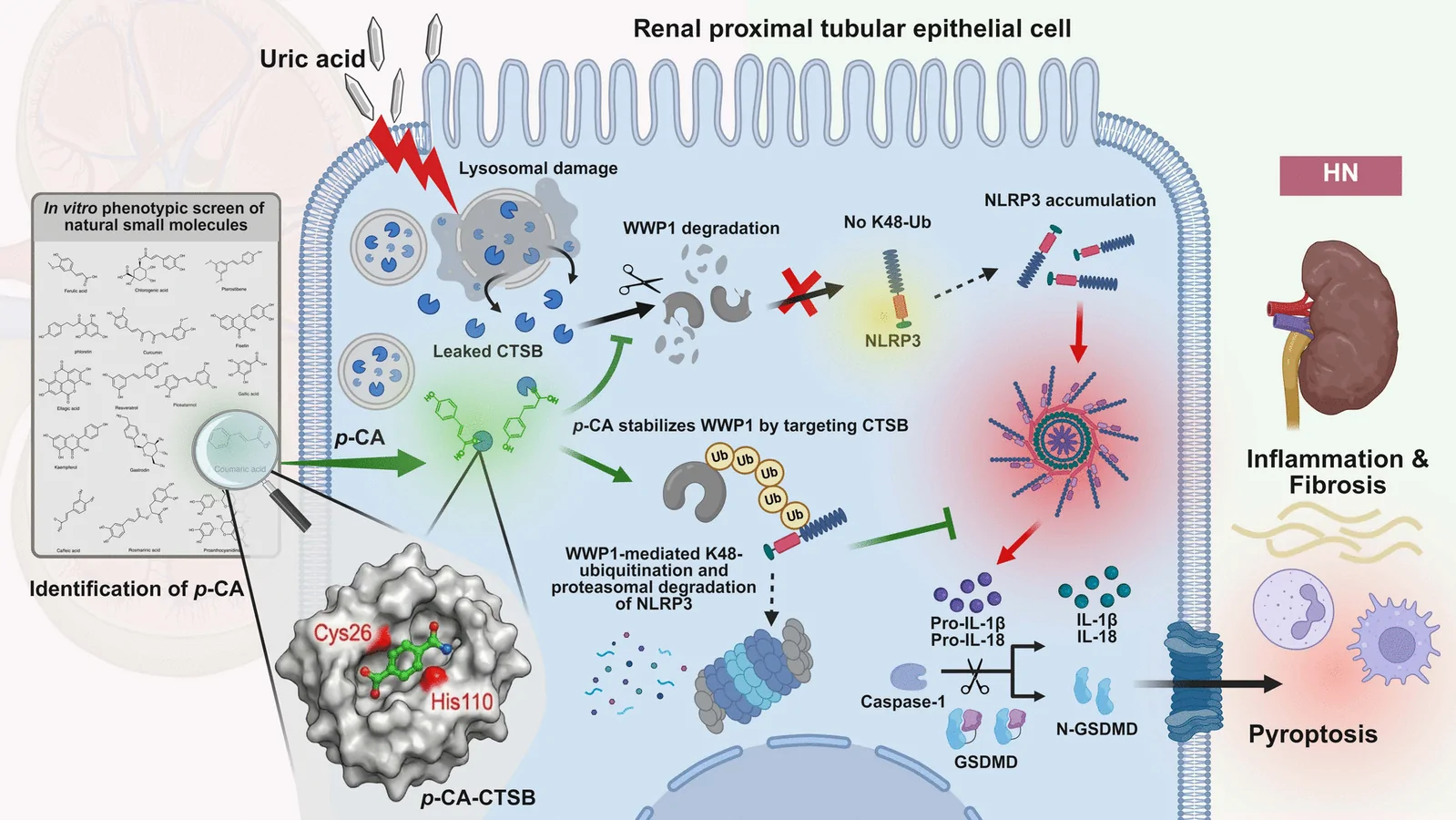

**Supplementary Information:**

The online version contains supplementary material available at https://doi.org/10.1186/s10020-026-01544-y.

## Introduction

Hyperuricemic nephropathy (HN) is a progressive metabolic and inflammatory disorder fundamentally driven by the dysregulated homeostasis of purine metabolism and a rising global prevalence of hyperuricemia (Dehlin et al. 2020; Hyndman et al. 2016). Chronic hyperuricemia leads to excessive tubular injury, interstitial inflammation, and eventual tubulointerstitial fibrosis, which may advance to kidney failure (Kang and Nakagawa 2005; Lusco et al. 2017; Srivastava et al. 2018). A central molecular event driving this disease progression is the aberrant activation of the NLR family pyrin domain containing 3 (NLRP3) inflammasome, which serves as a key pathogenic mediator (Latz et al. 2013; Su et al. 2020). Emerging experimental evidence indicates that uric acid (UA) acts as a danger-associated molecular pattern (DAMP) to trigger NLRP3 assembly, culminating in caspase-1-dependent pro-inflammatory cytokine maturation (IL-1β, IL-18) and GSDMD-mediated pyroptosis (Li et al. 2019). Despite progress in understanding these molecular underpinnings, effective therapeutic options targeting intrarenal pathophysiology are severely limited. Notably, while current clinical management relies heavily on systemic UA-lowering agents (e.g., allopurinol and benzbromarone), these drugs exhibit limited efficacy in preventing HN progression (Badve et al. 2020) and frequently cause significant adverse effects (Pascart and Richette 2018). Given the current absence of clinically approved NLRP3 inhibitors (Coll et al. 2019), identifying novel therapeutic agents to specifically intercept the UA-mediated NLRP3 inflammatory axis has become a critical priority for HN management.

Natural products remain a cornerstone of drug discovery, with over 50% of FDA-approved small-molecule drugs originating from natural scaffolds (Newman and Cragg 2020). Among these, phenolic acids—abundant in fruits, vegetables, and cereals—have attracted significant interest for their diverse bioactivities (Xu et al. 2017). Despite their broad biological activities, the specific exploration of phenolic compounds capable of directly targeting the pathogenic mechanisms of HN remains a critical unmet need. *p*-coumaric acid (*p*-CA), a phenolic compound ubiquitous in plant-based diets, exhibits a broad spectrum of bioactivities, encompassing antioxidant, anti-inflammatory, immunomodulatory, anti-tumor, cardioprotective, and neuroprotective properties (Chen et al. 2024; Malik and Dhiman 2022). While *p*-CA has demonstrated renoprotective potential in obstructive nephropathy via M2 macrophage modulation (Yao et al. 2025) and in cadmium toxicity through antioxidant restoration (Navaneethan and Rasool 2014), the specific therapeutic efficacy, precise molecular targets, and underlying mechanisms by which it ameliorates urate-induced renal injury remain largely unexplored.

Although diverse cellular stressors—such as ion efflux, mitochondrial dysfunction, and lysosomal destabilization (Swanson et al. 2019)—are known to activate NLRP3 assembly, the precise molecular events initiating this self-amplifying loop of chronic inflammation specifically in the context of HN remain poorly defined. Cathepsin B (CTSB), a lysosomal cysteine protease with potent proteolytic activity, has been demonstrated to modulate inflammation, apoptosis, extracellular matrix homeostasis, cancer, and endothelial function (Cocchiaro et al. 2017). Although prior studies have implicated CTSB in the activation of the NLRP3 inflammasome (Elliott and Sutterwala 2015), the exact mechanistic link coupling CTSB leakage to NLRP3 inflammasome activation in HN pathology has yet to be elucidated. Moreover, post-translational modification, particularly E3 ligase-mediated ubiquitination, are increasingly recognized as important regulators of NLRP3 inflammasome stability and activity (Beesetti 2025; Lopez-Castejon 2020). Recent studies have identified WW domain containing E3 ubiquitin protein ligase 1 (WWP1) as a critical executioner of NLRP3 degradation (Gao et al. 2024; Zhang et al. 2022; Zeng et al. 2024). However, whether the stability and NLRP3-regulatory function of the endogenous WWP1 pool are compromised by cytosolic CTSB under the metabolic stress of hyperuricemia remains poorly characterized.

To address these critical knowledge gaps and identify novel pharmacological leads, we performed a targeted in vitro phenotypic screen using a focused library of 16 bioactive phenolic compounds against UA-induced NLRP3 inflammatory responses. Through this strategy, we identified *p*-CA as a potent candidate for HN intervention. Mechanistically, our data support a post-lysosomal regulatory model in which *p*-CA engages the CTSB active-site/occluding-loop region and preserves WWP1 protein abundance in a CTSB activity-sensitive manner. This model links lysosomal CTSB leakage to impaired WWP1-dependent NLRP3 clearance, while recognizing that the precise molecular mechanism by which CTSB activity reduces WWP1 stability remains to be fully defined. By rescuing this WWP1-NLRP3 ubiquitination-clearance axis, *p*-CA effectively mitigates pyroptosis, inflammatory cascades, and subsequent renal fibrosis. Furthermore, cohort-level clinical analyses supported the relevance of CTSB dysregulation in hyperuricemia-associated CKD, providing a molecular rationale for further evaluating *p*-CA as a CTSB-directed pharmacological candidate in urate-induced renal injury.

## Material and methods

### Antibodies and reagents

*p*-Coumaric acid (T2863) with a purity of ≥ 98% and CA-074Me (T3420) were obtained from Targetmol (Boston, MA, USA); uric acid (U2625), cycloheximide (C7698), chloroquine (C6628) and anti-α-SMA (A2547) antibodies were obtained from Sigma-Aldrich (St. Louis, MO, USA); MG-132 (HY-13259) and acridine orange hydrochloride (HY-101879) were obtained from MCE (Monmouth Junction, NJ, USA); adenine (CA1221) was obtained from Coolaber (Beijing, China); and potassium oxonate (P831461) was obtained from Macklin (Shanghai, China). Recombinant human CTSB protein (10,483-H08H) was obtained from SinoBiological (Beijing, China). The Cathepsin B Inhibitor Screening Assay Kit (79,590–1), Cathepsin L Inhibitor Screening Assay Kit (79,591–1), and Cathepsin S Inhibitor Screening Assay Kit (79,588–1) were purchased from BPS Bioscience (San Diego, CA, USA). Anti-ASC (ab180799), anti-caspase-1 (ab179515), anti-IL-18 (ab243091, ab191860), anti-IL-1β (ab254360), anti-Fibronectin (ab45688), anti-Collagen I (ab260043), anti-Collagen III (ab184993), anti-CTSB (ab125067, ab214428), anti-LAMP1 (ab289548), anti-GAPDH (ab8245), anti-AQP-1 (ab168387) and anti-MPO (ab208670) antibodies were obtained from Abcam (Cambridge, MA, USA). Anti-pro-IL-1β (bs-0812R) antibody was obtained from Bioss (Woburn, MA, USA); anti-GSDMD (sc-393581), anti-CTSB (sc-365558) and anti-CD68 (sc-20060) antibodies were obtained from Santa Cruz Biotechnology (Dallas, TX, USA); anti-WWP1 (28,689–1-AP), anti-TRIM31 (12,543–1-AP), anti-IL-6 (HZ-1019), anti-MCP-1 (26,161–1-AP) and anti-TNF-α (17,590–1-AP) antibodies were obtained from Proteintech Group (Rosemont, IL, USA); and anti-NLRP3 (GB114320) antibody was obtained from Servicebio (Wuhan, China). The anti-NLRP3 (AG-20B-0014) antibody was obtained from AdipoGen Life Sciences (San Diego, CA, USA); the anti-MARCHF7 (H00064844-M01) antibody was obtained from Novus Biologicals (Centennial, CO, USA); and the anti-ubiquitin (3936), anti-K48-linkage specific polyubiquitin (8081), and anti-K63-linkage specific polyubiquitin (5621) antibodies were obtained from Cell Signaling Technology (Danvers, MA, USA).

### Human samples

Normal control tissues were obtained from the macroscopically healthy renal cortex of kidney donors with no history of renal pathology. Patients in the hyperuricemia-associated CKD cohort were enrolled based on a documented history of hyperuricemia or gout, after excluding other primary glomerular or tubular nephropathies. Serum urate levels were variable due to ongoing, individualized urate-lowering therapy. Although urate crystal deposition was not universally detected in biopsies—a phenomenon attributed to focal crystal distribution and crystal dissolution during standard histological processing—the established clinical history and the absence of alternative etiologies supported the diagnosis for this study. Comprehensive clinical parameters for all enrolled patients are detailed in Additional file 1: Table S1. From these patients, renal biopsy specimens, along with corresponding blood and urine samples, were procured for analysis. The collection and use of human specimens were approved by the Ethics Committee of Shanghai Tongren Hospital, Shanghai Jiao Tong University School of Medicine (Approval Number: K2025-047–01). Informed consent was obtained from all participants, and the study was conducted in accordance with the Declaration of Helsinki.

### HN rat model and *p*-CA administration

Male Sprague–Dawley rats (8 weeks old, 200 ± 20 g) were obtained from Hangzhou Ziyuan Experimental Animal Technology Corp. Ltd (Hangzhou, China). Rats were kept in a specific-pathogen-free (SPF) environment with regulated temperature (20–23 °C), humidity (50–70%), and a standard 12 h light/dark cycle. They received sterilized food and water freely, with a 1-week acclimation period before experiments.

A combination of potassium oxonate (1.5 g/kg/d) as well as adenine (0.1 g/kg/d) dissolved in 0.5% Carboxymethyl Cellulose Sodium (CMC-Na) was administered daily by oral gavage for 3 weeks to establish the HN rat model (Liu et al. 2015). To assess the efficacy of *p*-CA in HN rats, experimental doses of 50 and 100 mg/kg were selected based on previous studies and administered via oral gavage (Konishi et al. 2004; Neog et al. 2017). Vehicle control animals were given an isovolumetric 0.5% CMC-Na solution via identical orogastric administration protocols as the treatment groups. The pharmacological effect of CTSB inhibition was evaluated through daily intraperitoneal administration of CA-074Me (50 mg/kg) to HN rats for 3 weeks as a mechanistic positive control. After the experimental period, rats were anesthetized and euthanized, and their kidneys were immediately procured. Urinary microalbumin excretion was quantified from 24 h urine specimens collected using metabolic cages. Aspartate aminotransferase (AST), serum creatinine (Scr), blood urea nitrogen (BUN), alanine aminotransferase (ALT), and serum uric acid (SUA) were measured using an automated biochemical analyzer. All animal experiments were approved by the Ethics Committee of Tongren Hospital affiliated with Shanghai Jiao Tong University (Approval Number: A2023-043–01), and complied with the National Institutes of Health Guide for the Care and Use of Laboratory Animals.

### Cell culture and drug treatments

The human proximal tubule cell line HK-2 was obtained from the Cell Bank of the Chinese Academy of Sciences (Shanghai, China). Cells were maintained in an incubator at 37 °C with 5% CO_2_ using Dulbecco’s Modified Eagle Medium/F12 (1:1) complete medium, which was supplemented with 1% penicillin/streptomycin and 10% fetal bovine serum (FBS). Prior to experiments, HK-2 cells were synchronized by 24 h incubation in low-serum DMEM (0.5% FBS). Cells were treated with 800 μM UA for 48 h, with or without *p*-CA (50, 100, and 200 μM). For CTSB inhibition, UA-induced HK-2 cells were treated with CA-074Me (15 μM) for 48 h. For protein degradation assays, cells were treated with the protein synthesis inhibitor cycloheximide (CHX, 50 μg/mL) for 0, 2, 4, or 8 h before harvesting to determine protein half-life. To delineate specific degradation pathways, cells were co-incubated with the proteasome inhibitor MG-132 (10 μM) or the autophagy-lysosome inhibitor chloroquine (CQ, 20 μM) during the final 6 h of the 48-h UA stimulation prior to cell lysis.

Primary human proximal tubular epithelial cells (PTECs) were obtained from ScienCell (San Diego, CA, USA) and cultured in specialized epithelial cell medium (EpiCM, ScienCell) with 5% FBS, 1% penicillin/streptomycin, and 1% epithelial cell growth supplement. PTECs were subjected to the same UA and *p*-CA treatment regimen as described for HK-2 cells. All experiments were performed in at least three independent biological replicates.

### Drug screening assay

We selected 16 phenolic natural compounds based on a literature review of their reported anti-inflammatory properties. All compounds (purity ≥ 98%) were dissolved in DMSO to prepare stock solutions. A screening concentration of 200 μM was selected in accordance with established literature for the anti-inflammatory effects of phenolic derivatives (Kinra et al. 2021). After 24 h of incubation in 96-well plates at a density of 1 × 10^4^ cells/well, HK-2 cells were co-treated with 800 μM UA and 200 μM of each phenolic compound. NLRP3 inflammasome activation was assessed by measuring IL-1β secretion in supernatants using ELISA kits (ab214025, Abcam, USA). The compounds screened (Additional file 1: Table S2) included: Chlorogenic Acid, Caffeic Acid, Phloretin, Ellagic Acid, *p*-Coumaric Acid (*p*-CA), Curcumin, Ferulic Acid, Gallic Acid, Fisetin, Resveratrol, Piceatannol, Gastrodin, Kaempferol, Rosmarinic acid, Proanthocyanidins, and Pterostilbene.

### Cell viability assay

Cell viability was evaluated by the CCK-8 colorimetric assay (C0042, Beyotime, Shanghai, China). HK-2 cells were plated in 96-well plates at 1.0 × 10^4^ cells per well. Once cells reached approximately 70% confluence, they were exposed to UA at different concentrations (0, 200, 400, 800, and 1200 μM) for 48 and 72 h or *p*-CA at five distinct concentrations (0, 50, 100, 200, and 500 μM) for 48 h at 37 °C. To evaluate the cytoprotective potential of *p*-CA against UA-induced toxicity, HK-2 cells were co-treated with 800 μM UA and varying concentrations of *p*-CA.

### siRNA and plasmids transfection

CTSB-specific siRNA (si-CTSB), specific siRNAs targeting E3 ubiquitin ligases (si-TRIM31, si-MARCHF7, and si-WWP1), and scrambled negative control siRNA (NC siRNA) were designed and synthesized by Ribobio (Guangzhou, China). The specific sequences for all siRNAs used are listed in Additional file 1: Table S3. HK-2 cells were transfected at 50%–60% confluence using Lipofectamine RNAiMAX (13,778,150, Invitrogen, Carlsbad, CA, USA) according to the manufacturer’s instructions.

For plasmid transfection, HK-2 cells were seeded in 60-mm dishes and grown to 70%–80% confluence. Cells were then transfected with 5 μg of plasmid DNA (CTSB-WT or mutants: Cys26A, Gln23A, His110A, or Gly198A) using a DNA transfection reagent according to the manufacturer’s protocol. Following a 10-h transfection period, the cells were subjected to subsequent treatments.

### CTSB activity assay

CTSB activity in cell lysates or tissue homogenates was examined using a commercially available fluorometric assay kit (ab65300, Abcam, Cambridge, MA, USA). Fluorescence readings were measured with a Varioskan LUX microplate reader (Thermo Fisher Scientific, Waltham, MA, USA).

### Isolation of subcellular fractions

The Minute™ Lysosome Isolation Kit (LY-034, Invent Biotechnologies, Plymouth, MN, USA) was used to separate cytoplasmic and lysosome fractions. Protein concentrations were determined by the BCA assay, and the subcellular redistribution of CTSB was analyzed by Western blot.

### ELISA

The concentrations of IL-1β (ab214025, Abcam), IL-18 (KE00193, Proteintech), and CTSB (ab119584, Abcam) in clinical specimens were measured using specific ELISA kits according to the manufacturers’ instructions. To account for variations in urine dilution, we normalized urinary concentrations to creatinine levels and expressed them as picograms per milligram of creatinine (pg/mg Cr).

### Immunoblot analysis

Cellular and tissue samples were lysed using ice-cold RIPA buffer, supplemented with phosphatase and protease inhibitors (Beyotime, Shanghai, China). The lysates were centrifuged at 12,000 × *g* for 15 min at 4 °C, and the protein concentration of the supernatants was quantified using Beyotime’s BCA assay kit (Shanghai, China). Subsequently, proteins were separated by SDS-PAGE and electrotransferred onto PVDF membranes (Millipore, Burlington, MA, USA). Following a 1-h block in 5% non-fat milk at room temperature, the membranes were incubated overnight using primary antibodies at 4 °C, along with HRP-conjugated secondary antibodies at room temperature for 1 h. Protein signals were developed with ECL substrate (MeilunBio, Dalian, China), imaged under non-saturating conditions (Tanon, Shanghai, China), and quantified by ImageJ with GAPDH normalization.

### RNA extraction and qRT-PCR analysis

Total RNA was extracted using TRIzol reagent (Invitrogen, Carlsbad, CA, USA) according to the manufacturer’s protocol. Takara’s reverse transcription system kit (Kusatsu, Japan) was used to generate cDNA for mRNA quantification. qRT-PCR was performed using SYBR Green PCR Master Mix (Vazyme, Nanjing, China) on a Bio-Rad CFX96 Real-Time PCR System (Hercules, CA, USA). Quantification of relative mRNA expression employed the 2^−ΔΔCt^ method, normalized to GAPDH. The primer sequences (5’−3’) used during qRT-PCR are as follows:

- Human-CTSB-F: TGTTCTTGCGACTCTTGG
- Human-CTSB-R: GAAGGTTGACGAGGATGAC
- Human-NLRP3-F: ATGGGTTTACTGGAGTACCTTC
- Human-NLRP3-R: CTGTCTTCAATGCACTGGAATCTG
- Human-WWP1-F: TGAACAGTGGCAATCTCAGCGG
- Human-WWP1-R: CTGGTGGCAAAGGTCCATAAGG
- Human-GAPDH-F: ACAACTTTGGTATCGTGGAAGG
- Human-GAPDH-R: GCCATCACGCCACAGTTTC

### Histology and immunohistochemical staining

Kidney tissues fixed in 4% paraformaldehyde were paraffin-embedded and sectioned (4 μm). Sections were stained with H&E, Masson’s trichrome, and Periodic acid-Schiff (PAS). In each group, 30 random non-overlapping fields were analyzed to quantify the fibrotic area (Masson’s trichrome-positive staining) and the mean tubular damage score, assessed on H&E-stained sections according to criteria including tubular dilation, luminal necrotic debris, and epithelial necrosis. Tubular injury scores were quantified as follows: 0 = normal; 1 = < 25%; 2 = 25% to 50%; 3 = 50% to 75%; and 4 = > 75%. For immunohistochemical staining, kidney sections were blocked for 1 h using 5% bovine serum albumin at room temperature before incubating them with primary antibodies against caspase-1, NLRP3, α-smooth muscle actin (α-SMA), CTSB, CD68, as well as myeloperoxidase (MPO) overnight at 4 °C. Sections were incubated at room temperature for 1 h with HRP-conjugated secondary antibodies. Immunoreactivity was visualized using diaminobenzidine, and the positively stained area was quantified using Image Pro-Plus software (Silver Spring, MD). To ensure objective quantification, images were processed with a standardized thresholding algorithm applied consistently across all experimental groups.

### RNA-seq transcriptomic assay

Thermo Fisher Scientific’s Trizol reagent (Carlsbad, CA, USA) was used to extract total RNA from the control or HN group kidneys (n = 3). Paired-end sequencing was then performed on the Illumina Novaseq™ 6000 platform at LC-BIO Bio-tech Ltd (Hangzhou, China), following the manufacturer’s suggested protocol. Analysis of differentially expressed genes was conducted with the Limma R package (V3.58.1). The absolute fold change ≥ 2 and a false discovery rate < 0.05 were used as the criteria for differential genes. Volcano plots and heatmaps of the significant differentially expressed genes were generated using R software (ggplot2 version 3.5.0 and pheatmap version 1.0.12).

### Integrated analysis of GEO and single-cell RNA-seq datasets

R software was used to analyze the GSE190205 and GSE262687 datasets, which were obtained from the Gene Expression Omnibus (GEO) database. Differentially expressed genes were identified using the Limma R package (V3.58.1). Significance was defined as an absolute fold change ≥ 2 and a false discovery rate < 0.05.

The Kidney Precision Medicine Project Platform was used to analyze the single-cell RNA-seq data. To delineate CTSB-positive cell localization in CKD patients, we employed Uniform Manifold Approximation and Projection (UMAP) visualization of renal cell subgroups.

### Immunofluorescence staining

Paraffin-embedded kidney tissues were sectioned at 4 μm and subjected to standard deparaffinization and rehydration. After antigen retrieval and blocking, sections were incubated with primary antibodies against IL-1β, NLRP3, CTSB, Gasdermin D (GSDMD), and IL-18, followed by incubation with the corresponding fluorescent secondary antibodies (Invitrogen, Carlsbad, CA, USA). For cell culture, HK-2 cells were plated on confocal dishes and subjected to a 15-min fixation in 4% paraformaldehyde. Following permeabilization in 0.1% Triton X-100, the cells were blocked with a solution containing 3% goat serum plus 3% BSA (1 h, room temperature). Tissue sections and cells were exposed to primary antibodies against the targets overnight at 4 °C. The samples were subsequently treated with appropriate secondary antibodies, and 4',6-diamidino-2-phenylindole (DAPI) was used for nuclear counterstaining. Fluorescence images were captured by confocal microscopy (Leica, Wetzlar, Germany) and quantified using ImageJ software.

### Acridine orange (AO) staining

Lysosomal membrane permeability (LMP) was assessed in HK-2 cells with or without UA treatment (48 h). Cells were stained with acridine orange (MCE, Monmouth Junction, NJ, USA) (5 μM) for 15 min at 37 °C. Following PBS washes, cellular fluorescence was visualized using a Leica confocal microscope (488 nm excitation), with dual emission detection (505–570 nm/615–754 nm). In intact lysosomes, AO accumulates as protonated oligomers emitting red fluorescence; upon LMP, it leaks into the cytosol, deprotonates, and fluoresces green. Quantitative analysis was performed using ImageJ software.

### Coimmunoprecipitation (Co-IP) and NLRP3 ubiquitination assay

For co-immunoprecipitation analysis, HK-2 cells treated with UA and *p*-CA for 48 h were lysed in immunoprecipitation buffer (Beyotime, Shanghai, China) supplemented with protease and phosphatase inhibitor cocktails. After centrifugation at 12,000 × g for 15 min at 4 °C, a portion of the supernatant was boiled in SDS loading buffer as the input control. The remaining cell lysates were incubated with anti-CTSB or normal IgG antibodies overnight at 4 °C, followed by incubation with Protein A/G magnetic beads (Yeasen, Shanghai, China) for 4 h at 4 °C. The immunoprecipitated complexes were washed five times with ice-cold lysis buffer, eluted by boiling, and subjected to immunoblot analysis.

For the NLRP3 ubiquitination assay, cells were pre-treated with 10 μM MG-132 for 6 h prior to harvesting. Cells were then directly lysed in denaturing lysis buffer (PBS containing 50 mM Tris–HCl at pH 7.5, 1% SDS, 5 mM N-ethylmaleimide [NEM], and protease inhibitor cocktail). The lysates were boiled for 10 min to dissociate noncovalent protein interactions, and subsequently diluted ten times with lysis buffer (PBS containing 50 mM Tris–HCl at pH 7.5, 1% Triton X-100, 5 mM NEM). After centrifugation (12,000 × g for 15 min at 4 °C), the supernatants were immunoprecipitated with an anti-NLRP3 antibody overnight at 4 °C and captured by magnetic beads. The purified immune complexes were finally immunoblotted to detect total NLRP3, pan-ubiquitin, and specific ubiquitin linkages (K48 and K63).

### Network pharmacology analysis

To investigate potential targets of *p*-CA, an exploratory network pharmacology approach was employed using the Traditional Chinese Medicine Systems Pharmacology (TCMSP) Database, SuperPRED, Swiss Target Prediction, PharmMapper, ChemBL, and STITCH. Disease-related targets were obtained from the DisGeNET, DrugBank, GeneCards, the Pharmacogenomics Knowledgebase (PharmGKB), the Therapeutic Target Database (TTD), and the Online Mendelian Inheritance in Man (OMIM) databases, using keywords “hyperuricemic nephropathy”, “uric acid nephropathy”, or “gouty nephropathy”. Data integration was performed to identify candidate hub targets among those predicted for *p*-CA, the HN-associated targets, and targets from RNA-sequencing data. These common targets were visualized using the Venn diagram package (v1.12), and the ggvenn package (v0.1.10). The biological functions of the common targets were analyzed by performing KEGG pathway enrichment analysis using the clusterProfiler package (v4.10), using a *P*-value < 0.05 as the threshold. A protein–protein interaction network of the shared targets was constructed using the STRING database with a combined score > 0.4 as the cutoff. Biological processes were investigated using Gene Ontology (GO) analysis. Candidate hub targets were prioritized using the MCODE and cytoHubba plug-ins within Cytoscape. The cytoHubba plug-in (version 3.10.1) was specifically used with 12 algorithms: Density of Maximum Neighborhood Component (DMNC), Maximal Clique Centrality (MCC), Degree, Maximum Neighborhood Component (MNC), BottleNeck, Edge Percolated Component (EPC), Closeness, Eccentricity, Betweenness, Radiality, Clustering Coefficient, and Stress.

### Molecular docking

We obtained the crystal structure of human CTSB (as the receptor) and *p*-CA (as the ligand) from the Protein Data Bank (ID: 8B4T) and the PubChem database, respectively. To ensure structural transparency, all residue numbering used in this manuscript strictly corresponds to the utilized crystal structure (PDB ID: 8B4T). The functional roles and structural mapping of these key residues are explicitly detailed in Additional file 1: Table S4. The receptor and ligand molecules were preprocessed using AutoDock MGL Tools to prepare the active sites of the receptor protein preceding the molecular docking analysis. We employed AutoDock Vina (version 1.2.2) for molecular docking to predict potential binding poses and identify interaction hotspots. The optimal docking complexes were subsequently selected for visualization using PyMol software (version 2.5.2).

### Molecular dynamics simulation (MDS)

To examine the binding interaction between *p*-CA and CTSB, AMBER 18 software was utilized to perform MDS. Before simulation, the charge of the small molecule was calculated using the Hartree–Fock self-consistent field method with a 6-31G basis set and the antechamber module in Gaussian 09. Proteins and small molecules were processed using the ff14SB protein force field and the General Amber Force Field version 2, respectively (Maier et al. 2015). Hydrogen atoms were added to each system using the LEap module. In the system, a truncated octahedral TIP3P water box was positioned 10 Å away. To achieve charge neutrality, Na^+^ and Cl^−^ ions were introduced. Energy minimization was performed using 2,500 steps of steepest descent, followed by conjugate gradient optimization. We conducted simulations using both the isothermal-isobaric ensemble and the isothermal-isochoric ensemble. Finally, MDS was performed for a total duration of 100 ns, and the relevant results were analyzed using the software’s built-in tools. The binding free energy of the protein-small molecule complex was determined by applying the Molecular Mechanics/Generalized Born Surface Area (MM/GBSA) method to MD trajectories collected from the 90–100 ns interval.

### High performance liquid chromatography-tandem mass spectrometry (HPLC–MS/MS) analysis

HK-2 cells were treated with 200 μM *p*-CA or vehicle for 48 h. After PBS washing to remove extracellular compounds, cells were lysed in 50% methanol/water by three freeze–thaw cycles followed by ultrasonication to ensure complete intracellular metabolite extraction. The supernatants were analyzed using an ACQUITY UPLC Premier system coupled to a Xevo TQ-Absolute triple quadrupole mass spectrometer (Waters, Milford, MA, USA) operating in multiple reaction monitoring mode. Employing a Waters BEH C18 column (100 × 2.1 mm, 1.7 μm) at 45 °C, separation was achieved using a mobile phase of 0.1% aqueous formic acid (A) and acetonitrile (B). All samples (1 μL injection volume) were separated using an isocratic elution method with a mobile phase composition of 20% A and 80% B at 0.4 mL·min^−1^.

Target quantification was carried out by monitoring the precursor-to-product ion transition from m/z 163 to 119. Key mass spectrometric parameters were: electrospray voltage, 1 kV; capillary temperature, 150 °C; and ion transfer tube temperature, 450 °C. Collision-induced dissociation was performed at 26 eV, and the cone voltage was optimized to 20 V.

### Cellular thermal shift assay (CETSA)

HK-2 cells cultured in 15-cm dishes were treated with DMSO or *p*-CA (200 μM) for 4 h. Following collection and PBS washes, cell pellets were resuspended in PBS supplemented with protease and phosphatase inhibitors, and equally aliquoted into 8 PCR tubes. Each sample was heated across a temperature gradient (37 °C, 42 °C, 47 °C, 52 °C, 57 °C, 62 °C, 67 °C, and 72 °C) for 3 min, followed by cooling at 25 °C for 3 min. Cells were then lysed via three freeze–thaw cycles using liquid nitrogen and a 37 °C water bath. The lysates were centrifuged at 20,000 × g for 20 min at 4 °C to pellet denatured proteins. The soluble protein fractions were collected and subjected to western blotting analysis. For mutant CETSA, HK-2 cells transfected with WT or mutant CTSB constructs were treated with DMSO or *p*-CA and subjected to the indicated thermal challenge before immunoblot detection of soluble CTSB.

### Surface plasmon resonance (SPR) assay

SPR analysis was conducted by a Biacore 8 k instrument (Cytiva, Marlborough, MA, USA). Recombinant His-labelled human CTSB Protein (HPLC-verified) was purchased from Sino Biological Inc. (Beijing, China). The CM5 sensor chip was covalently immobilized with the CTSB protein. All experiments were performed at 25 °C with a flow rate of 5 μL/min. The running buffer was phosphate-buffered saline containing 0.005% Tween-20 and 5% dimethyl sulfoxide (DMSO). The interaction between *p*-CA and the immobilized CTSB was measured. SPR data were analyzed with a 1:1 binding model by Biacore™ Insight Evaluation software to derive the following kinetic parameters: the association rate constant (*k*_*a*_), dissociation rate constant (*k*_*d*_), and equilibrium dissociation constant (*K*_*D*_).

### In vitro cathepsin enzymatic inhibition and selectivity assays

The in vitro inhibitory activity and selectivity of p-CA against CTSB, Cathepsin L (CTSL), and Cathepsin S (CTSS) were evaluated using fluorometric Cathepsin Inhibitor Screening Assay Kits (BPS Bioscience) according to the manufacturer’s instructions. Briefly, purified recombinant human enzymes were incubated with varying concentrations of p-CA (1–500 μM) prior to substrate addition. The steady-state fluorescence intensity was subsequently recorded to calculate the half-maximal inhibitory concentration (IC50) values.

### Circular dichroism analysis

A JASCO J-810 spectropolarimeter (Tokyo, Japan) was used to analyze secondary structural changes of CTSB by circular dichroism spectroscopy. Measurements were conducted at 25 °C using 0.5 mg/mL CTSB in 1 mm quartz cuvettes, with or without *p*-CA at a 1:1 molar ratio. The circular dichroism spectra were recorded at a scanning speed of 100 nm/min with 0.1 nm data intervals between 190 and 260 nm. Each spectrum represented the average of three scans. A baseline spectrum of *p*-CA, at an equivalent concentration without CTSB, was subtracted to isolate the contribution of the protein. The changes in the protein's secondary structure upon the addition of *p*-CA were analyzed using the Secondary Structure Estimation software.

### Statistical analysis

All statistical analyses were performed using GraphPad Prism (v10.0). Data were presented as mean ± SEM unless otherwise specified. Normality and homogeneity of variance were assessed using the Shapiro–Wilk test and the Brown-Forsythe/F-test, respectively. For comparisons between two groups, we applied the Student’s t-test (if normality and equal variance assumptions were met) or the Mann–Whitney U test (for non-normally distributed data). Multiple-group comparisons were analyzed by one-way ANOVA followed by Tukey’s multiple comparisons test (parametric) or Kruskal–Wallis test with Dunn’s correction (non-parametric), as appropriate. Dual-factor and longitudinal data (e.g., rat body weight) were analyzed via two-way ANOVA followed by Bonferroni’s multiple comparisons. Dose-dependent trends were confirmed using simple linear regression. Non-linear regression was utilized to fit dose–response curves for enzymatic target selectivity and to analyze quantitative CETSA melt curves (using a Boltzmann sigmoidal model, with Δ*T*_*m*_ shifts determined by the extra sum-of-squares F-test). Clinical correlation analyses were evaluated using Spearman’s rank-order correlation to accommodate the clinical cohort size. The threshold for statistical significance was set at *P* < 0.05.

## Results

### Targeted screening reveals *p*-CA blunts UA-induced NLRP3 activation

**Fig. 1:**
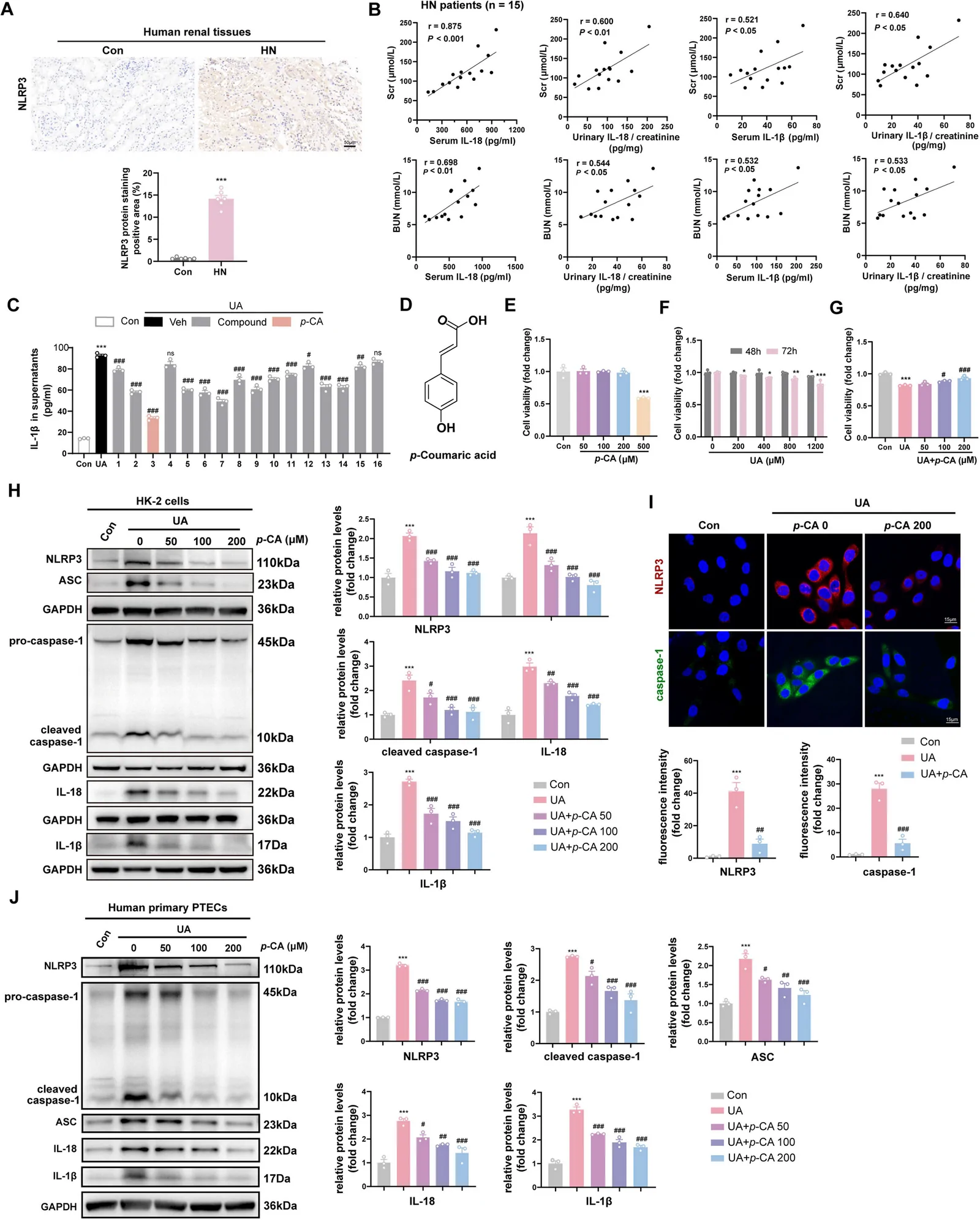
Targeted screening reveals *p*-CA blunts UA-induced NLRP3 activation. **A** Representative immunohistochemical staining of NLRP3 in renal tissues from healthy controls (Con) and the hyperuricemia-associated CKD cohort, with the histogram below showing the quantification of the positive area (*n* = 6). Scale bar = 50 μm. **B** Spearman's correlation analysis showing the relationship between renal function markers (Scr, BUN) and inflammatory cytokines (Serum/Urinary IL-18, IL-1β) in the hyperuricemia-associated CKD cohort (*n* = 15). **C** Screening of 16 natural phenolic compounds for their inhibitory effects on IL-1β secretion in supernatants of UA-stimulated HK-2 cells (measured by ELISA). *p*-CA is highlighted in red. **D** Chemical structure of *p*-CA. **E** Cell viability of HK-2 cells treated with increasing concentrations of *p*-CA (50–500 μM) for 48 h, assessed by CCK-8 assay. **F** Cytotoxicity of UA at various concentrations (200–1200 μM) on HK-2 cells at 48 and 72 h. **G** Protective effect of *p*-CA pretreatment (50, 100, and 200 μM) on cell viability in UA-stimulated HK-2 cells (48 h). **H** Western blot analysis of NLRP3 inflammasome components in HK-2 cells treated with UA (800 μM) and the indicated concentrations of *p*-CA for 48 h. **I** Immunofluorescence of NLRP3 (red) and caspase-1 (green), and fluorescence intensity measurement in HK-2 cells. Scale bar = 15 μm. **J** Western blot analysis showing the expression levels of NLRP3, cleaved caspase-1, ASC, IL-18, and mature IL-1β in human primary proximal tubular epithelial cells (PTECs) treated with UA and *p*-CA. For C-J, *n* = 3 independent experiments. All data are expressed as means ± SEM. Statistical significance was determined using an unpaired Student's t-test (for A), Spearman's correlation analysis (for B), and one-way analysis of variance (ANOVA) followed by Tukey’s multiple comparisons test (for C, E–J). A formal test for linear trend was confirmed using simple linear regression across the concentration gradients (all *P*_*trend*_ < 0.001). ns, not significant; ^*^P < 0.05, ^**^P < 0.01, and ^***^P < 0.001 *vs*. control group; ^#^*P* < 0.05, ^##^*P* < 0.01, and ^###^*P* < 0.001 *vs*. the UA group

To contextualize the relevance of NLRP3 inflammasome activation in our clinical cohort, we first examined kidney specimens from a hyperuricemia-associated CKD cohort. Immunohistochemical analysis showed increased NLRP3 immunoreactivity in the renal tissues of these patients compared to controls, predominantly localized to the renal tubules (Fig. 1A). Furthermore, serum and urinary levels of IL-18 and IL-1β—canonical markers of NLRP3 activation—exhibited significant positive correlations with clinical indicators of renal dysfunction, specifically serum creatinine (Scr) and blood urea nitrogen (BUN) (Fig. 1B). Together, these cohort-level data support the presence and clinical relevance of NLRP3-associated inflammatory activation in hyperuricemia-associated CKD, rather than presenting NLRP3 upregulation as a standalone novel finding. Furthermore, immunofluorescence co-staining with aquaporin-1 (AQP-1) in the kidneys of HN rats showed that this aberrant NLRP3 expression is predominantly localized to the proximal renal tubules (Additional file 1: Figure S1A).

Based on these cohort-level observations, we screened a library of 16 phenolic compounds with known anti-inflammatory properties (Additional file 1: Table S2) using UA-stimulated HK-2 cells to identify potential pharmacological candidates. Using IL-1β secretion as the primary readout for NLRP3 inflammasome activity, we observed varying degrees of inhibition among the compounds (Fig. 1C). Notably, compound 3, identified as* p*-CA (Fig. 1D), exhibited potent inhibitory activity in our screen, significantly reducing IL-1β levels. We next assessed the cytotoxicity of *p*-CA using a CCK-8 assay. *p*-CA did not significantly affect HK-2 cell viability at concentrations of 50, 100, and 200 μM (Fig. 1E). Conversely, UA treatment induced a time- and dose-dependent reduction in cell viability (Fig. 1F) and dose-dependently upregulated key NLRP3 components (NLRP3, caspase-1, IL-18) and fibrotic markers (Fibronectin, Collagen I, Collagen III), with maximal effects at 800 μM (Additional file 1: Figure S1B). Therefore, 800 μM UA for 48 h was used to establish the in vitro model for subsequent experiments. Importantly, *p*-CA treatment dose-dependently improved cell viability in the presence of UA (Fig. 1G).

Based on these results, *p*-CA concentrations of 50, 100, and 200 μM were selected for functional validation. Immunoblotting analysis revealed that *p*-CA dose-dependently attenuated the UA-induced upregulation of core NLRP3 components (NLRP3, ASC, cleaved caspase-1) and downstream cytokines (IL-18, IL-1β) in HK-2 cells (Fig. 1H). This suppressive effect was further corroborated by immunofluorescence analysis, which showed decreased expression and focal accumulation of caspase-1 and NLRP3 in *p*-CA-treated HK-2 cells (Fig. 1I). Crucially, the inhibitory effect of *p*-CA on the NLRP3 inflammatory cascade was successfully recapitulated in human primary proximal tubular epithelial cells (PTECs) (Fig. 1J). Notably, while UA stimulation upregulated precursor pro-IL-1β expression, *p*-CA treatment did not alter total pro-IL-1β protein levels, indicating that *p*-CA mainly suppresses post-translational maturation rather than upstream priming (Additional file 1: Figure S1C). Collectively, these findings identify *p*-CA as a potent suppressor of the NLRP3 inflammasome cascade in UA-stimulated renal tubular epithelial cells.

### *p*-CA ameliorates kidney injury and suppresses the NLRP3 inflammasome in HN rats

**Fig. 2:**
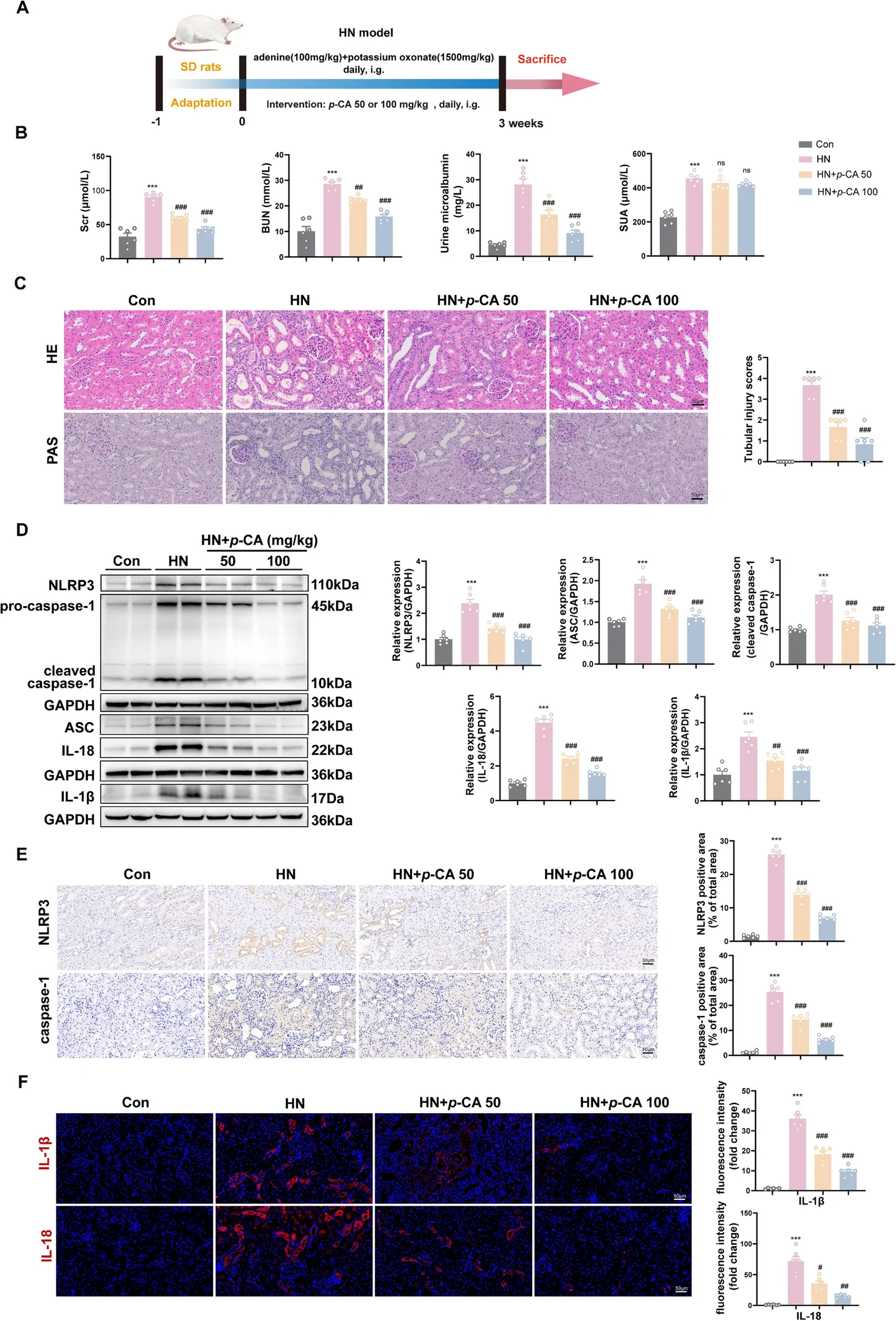
*p*-CA ameliorates kidney injury and suppresses the NLRP3 inflammasome in HN rats. **A** Schematic representation of HN rats with *p*-CA treatment. **B** Serum creatinine (Scr), blood urea nitrogen (BUN), urine microalbumin, and serum uric acid (SUA) levels across rat groups (*n* = 6). **C** Representative images of Hematoxylin and eosin (H&E) and Periodic acid-Schiff (PAS) staining of rat kidney sections, along with the quantification of tubular injury scores (*n* = 6). Scale bar = 50 μm. **D** Representative Western blots and corresponding quantification of NLRP3 pathway proteins in renal tissues from the indicated rat groups (*n* = 6). **E** Representative immunohistochemical images and quantification of the positive staining areas for NLRP3 and caspase-1 in rat kidney tissues (*n* = 6). Scale bar = 50 μm. **F** Representative immunofluorescence images and quantitative fluorescence intensity of IL-1β and IL-18 in rat kidney tissues (*n* = 6). Scale bar = 50 μm. All data are expressed as means ± SEM. Statistical significance was determined using one-way ANOVA followed by Tukey’s multiple comparisons test (for B–F). A formal test for linear trend was confirmed using simple linear regression across the concentration gradients (all *P*_*trend*_ < 0.001). ns, not significant; ^*^P < 0.05, ^**^P < 0.01, and ^***^P < 0.001 *vs*. control group; ^#^*P* < 0.05, ^##^*P* < 0.01, and ^###^*P* < 0.001 *vs*. the HN group

Using an established adenine/potassium oxonate-induced HN model (Fig. 2A) (Liu et al. 2015), we validated the pharmacological potential of p-CA in vivo. The model successfully recapitulated HN features, including severe renal impairment (elevated Scr, BUN, microalbuminuria) and hyperuricemia (Fig. 2B). Treatment with p-CA (50 and 100 mg/kg) significantly ameliorated renal dysfunction, as evidenced by marked reductions in Scr, BUN, and urine microalbumin. Intriguingly, despite these beneficial effects on renal parameters, p-CA treatment did not significantly alter serum uric acid (SUA) levels at either dose (Fig. 2B), suggesting a downstream renoprotective mechanism independent of systemic urate lowering. Consistent with this phenotype, Western blot analysis showed that the protein expression of major urate transporters (URAT1 and GLUT9) remained unchanged following p-CA administration (Additional file 1: Figure S2A).

Tubulointerstitial damage, encompassing interstitial fibrosis, tubular dilation, and atrophy, is a hallmark of HN pathology (Lusco et al. 2017). Histological assessment via PAS and H&E staining confirmed severe structural damage in the HN group compared to controls (Fig. 2C). Notably, *p*-CA treatment dose-dependently mitigated these pathological alterations, significantly improving the tubular injury scores derived from H&E staining (Fig. 2C). Collectively, these data indicate that *p*-CA alleviates pathological injury and preserves renal function in HN rats.

We further investigated whether the observed renoprotection was mechanistically linked to NLRP3 inhibition in vivo. Consistent with our findings in HK-2 cells, *p*-CA treatment dose-dependently reduced ASC, NLRP3, IL-18, cleaved caspase-1, and IL-1β expression in HN rat kidneys (Fig. 2D). Consistent with the in vitro findings, total pro-IL-1β accumulation in renal tissues remained unaffected by *p*-CA administration, supporting suppression of post-translational cytokine maturation in vivo (Additional file 1: Figure S2B). Immunohistochemical and immunofluorescence analyses further validated that elevated expression of caspase-1, NLRP3, IL-1β, and IL-18—primarily localized to the tubules and tubulointerstitium—was significantly attenuated by *p*-CA administration (Figs. 2E-F). Importantly, rats treated with *p*-CA did not exhibit overt signs of systemic toxicity during the 3-week experimental window, as evidenced by stable serum liver enzymes, including alanine aminotransferase (ALT) and aspartate aminotransferase (AST), and the absence of significant weight loss (Additional file 1: Figure S2C-D). Overall, these findings demonstrate that *p*-CA confers renoprotection by inhibiting NLRP3-mediated inflammation while showing short-term tolerability in this model.

### *p*-CA alleviates pyroptosis and inflammatory responses in vitro and in vivo

**Fig. 3:**
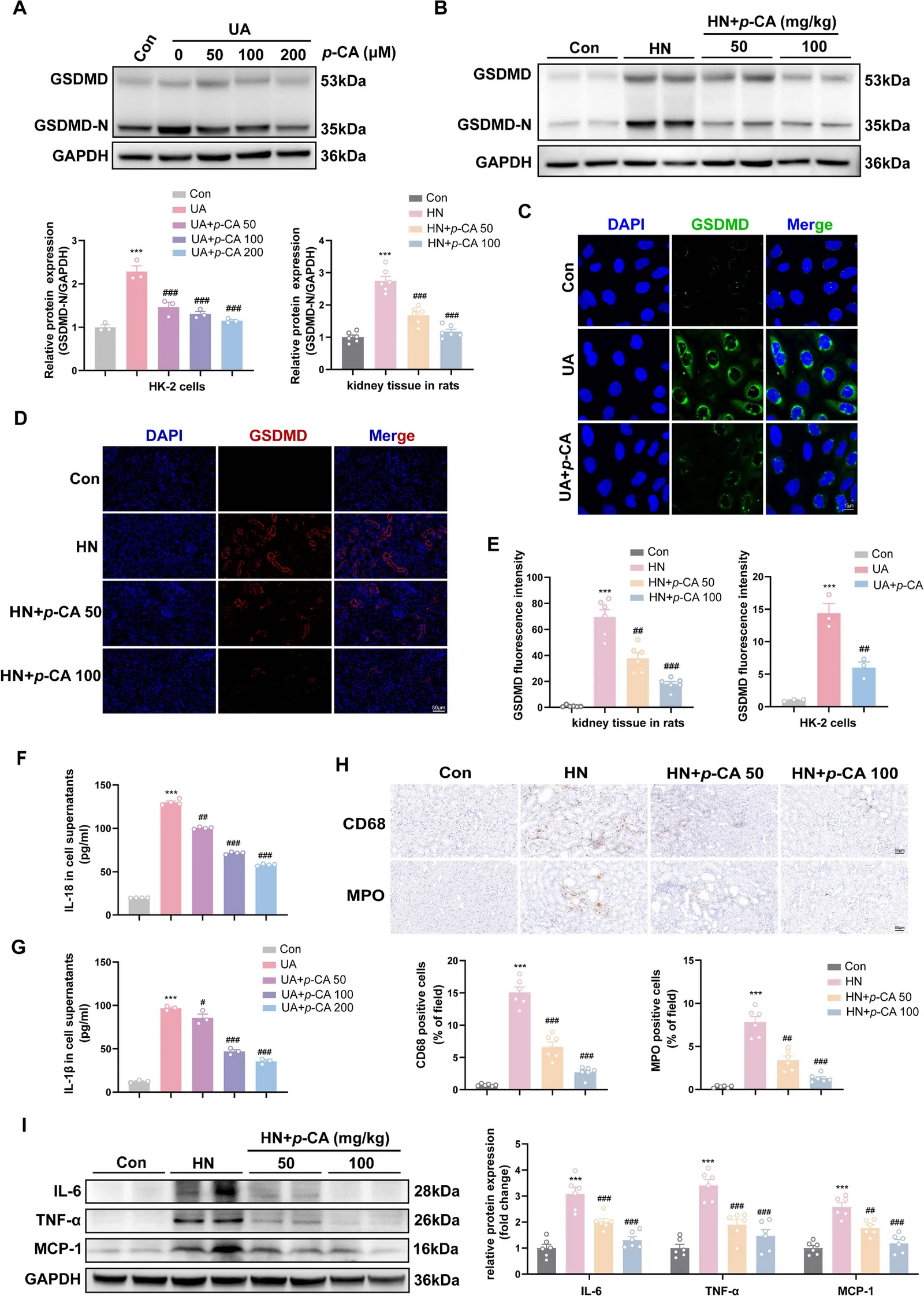
*p*-CA alleviates UA-induced pyroptosis and inflammatory responses in vitro and in vivo. **A**, **B** Representative Western blots and corresponding quantification of GSDMD and its active N-terminal fragment (GSDMD-N) in (**A**) UA-stimulated HK-2 cells treated with *p*-CA and (**B**) renal tissues from the indicated rat groups (*n* = 6). **C**, **D** Representative immunofluorescence images of GSDMD in (**C**) HK-2 cells (Scale bar = 15 μm) and (**D**) rat kidney tissues (Scale bar = 50 μm). **E** Quantitative analysis of GSDMD fluorescence intensity in rat kidney tissues (left, *n* = 6) and HK-2 cells (right). (**F**, **G**) ELISA quantification of (**F**) IL-18 and (**G**) IL-1β secretion in the supernatants of HK-2 cells. (H) Representative immunohistochemical images and quantification of macrophage (CD68) and neutrophil (MPO) infiltration in rat kidney sections (*n* = 6). Scale bar = 50 μm. **I** Representative Western blots and corresponding quantification of pro-inflammatory cytokines (IL-6, TNF-α, and MCP-1) in renal tissues from the indicated rat groups (*n* = 6). For in vitro assays (**A**, **C**, and **E**), *n* = 3 independent experiments. For F, n = 4 independent experiments. All data are expressed as means ± SEM. Statistical significance was determined using one-way ANOVA followed by Tukey’s multiple comparisons test (for all panels). A formal test for linear trend was confirmed using simple linear regression across the concentration gradients (all *P*_*trend*_ < 0.001). ns, not significant; ^*^P < 0.05, ^**^P < 0.01, and ^***^P < 0.001 vs. control group; ^#^*P* < 0.05, ^##^*P* < 0.01, and ^###^*P* < 0.001 vs. the UA or HN group

Caspase-1-mediated cleavage of GSDMD is a key execution step of pyroptosis, leading to membrane rupture and the release of inflammatory mediators (Huang et al. 2021). To establish the pathological relevance of this pathway in HN, we analyzed GEO datasets (GSE190205, GSE262687) and our internal RNA-seq data, both of which identified GSDMD as a significantly upregulated gene in HN renal tissues (Additional file 1: Figure S3A-B). Given the central role of the NLRP3/caspase-1/GSDMD axis in pyroptosis, we investigated whether *p*-CA could attenuate this pathway. In UA-stimulated HK-2 cells, human primary PTECs, and HN rats, levels of the active N-terminal cleavage product of GSDMD (GSDMD-N) were markedly elevated. *p*-CA treatment significantly and dose-dependently attenuated this UA-induced GSDMD cleavage both in vitro* and *in vivo (Figs. 3A-B). Furthermore, immunofluorescence analysis of HN rat kidneys demonstrated a significant upregulation of GSDMD, predominantly localized to damaged tubules, which was dose-dependently suppressed by *p*-CA administration (Figs. 3C-E).

GSDMD pore formation facilitates the release of mature IL-1β and IL-18, key drivers of the inflammatory cascade. We therefore evaluated the effect of *p*-CA on cytokine secretion in UA-stimulated HK-2 cells. ELISA results confirmed that UA stimulation induced a marked, dose-dependent release of IL-1β and IL-18 into culture supernatants (Additional file 1: Figure S3C). Notably, their secretion was attenuated in a dose-dependent manner by *p*-CA (Figs. 3F-G). Mechanistically, IL-1β and IL-18 promote immune-cell recruitment and amplify renal inflammation (Qi and Yang 2018). Consistent with this, immunohistochemical staining of HN kidney tissues revealed extensive infiltration of MPO-positive neutrophils and CD68-positive macrophages within the tubulointerstitium. *p*-CA administration remarkably reduced this immune cell infiltration in a dose-dependent manner (Fig. 3H). Subsequent immunoblot analysis of kidney lysates confirmed that the downstream pro-inflammatory cytokines MCP-1, TNF-α, and IL-6 were significantly upregulated in HN rats and effectively suppressed by *p*-CA treatment (Fig. 3I). Collectively, these findings indicate that *p*-CA alleviates pyroptosis and subsequent inflammatory responses both in vitro* and *in vivo*.*

### *p*-CA mitigates renal fibrosis in UA-induced renal tubular epithelial cells and HN rats

**Fig. 4:**
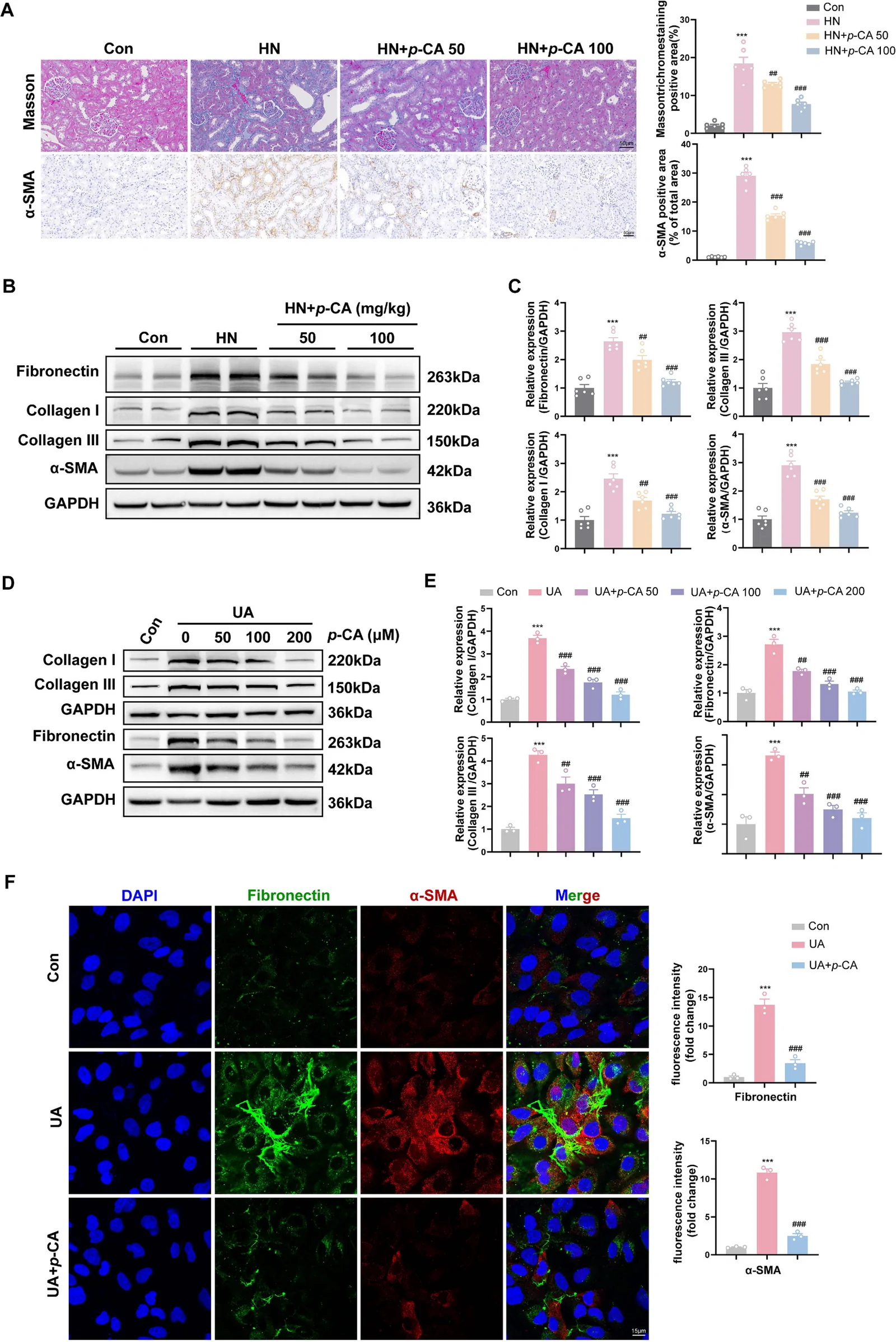
*p-*CA mitigates renal fibrosis in UA-induced renal tubular epithelial cells and HN rats. **A** Representative images of Masson's trichrome and immunohistochemical staining for α-SMA, along with the quantification of their respective positive areas in rat kidney tissues (*n* = 6). Scale bar = 50 μm. **B**, **C** Representative Western blots (**B**) and corresponding quantification (**C**) of fibrotic markers in renal tissues from the indicated rat groups (*n* = 6). (**D**-**E**) Representative Western blots (**D**) and corresponding quantification (**E**) of fibrotic markers in UA-stimulated HK-2 cells treated with the indicated concentrations of *p*-CA. (**F**) Representative immunofluorescence images and quantitative fluorescence intensity of Fibronectin (green) and α-SMA (red) in HK-2 cells. Scale bar = 15 μm. For in vitro assays (**D**–**F**), *n* = 3 independent experiments. All data are expressed as means ± SEM. Statistical significance was determined using one-way ANOVA followed by Tukey’s multiple comparisons test (for all panels). A formal test for linear trend was confirmed using simple linear regression across the concentration gradients (all *P*_*trend*_ < 0.001). ns, not significant; ^*^P < 0.05, ^**^P < 0.01, and ^***^P < 0.001 vs. control; ^#^*P* < 0.05, ^##^*P* < 0.01, and ^###^*P* < 0.001 vs. the UA or HN group

Activated NLRP3 inflammasome exacerbates renal inflammation and fibrosis in diverse kidney pathologies (Wang et al. 2013). Given that UA-induced NLRP3 inflammasome activation is crucial for driving the profibrotic phenotype of tubular cells and tubulointerstitial fibrosis (Braga et al. 2017; Romero et al. 2017), we further investigated the antifibrotic effects of *p*-CA in vitro and in vivo. Collagen deposition was evident throughout the renal tubulointerstitium, as shown by Masson’s trichrome staining. In contrast, *p*-CA treatment significantly attenuated fibrotic manifestations, as demonstrated by reduced blue-stained fibrotic areas (Fig. 4A). We next evaluated how *p*-CA affected α-SMA expression in HN rats, since this protein is an established marker of renal fibrosis and profibrotic cellular activation (Lin et al. 2008). While α-SMA expression was significantly elevated and predominantly localized in the tubulointerstitial region of HN kidneys, *p*-CA treatment significantly reduced the area of positive staining (Fig. 4A). Expression analysis of key fibrotic proteins (Collagen III, Collagen I, Fibronectin, and α-SMA) was subsequently performed by Western blotting. The HN rat kidneys showed high expression of these fibrotic markers (Figs. 4B-C), which were dose-dependently decreased by *p*-CA. Comparable results were observed in HK-2 cells and PTECs (Fig. 4D-E; Additional file 1: Figure S4A). Immunofluorescence staining of Fibronectin, α-SMA, Collagen III, and Collagen I further showed that *p*-CA reduced these profibrotic markers in HK-2 cells and PTECs (Fig. 4F; Additional file 1: Figure S4B). Collectively, these data support the antifibrotic effects of *p*-CA in UA-stimulated tubular epithelial cells and HN rats.

### Identification of CTSB as a functional target of ***p***-CA

**Fig. 5:**
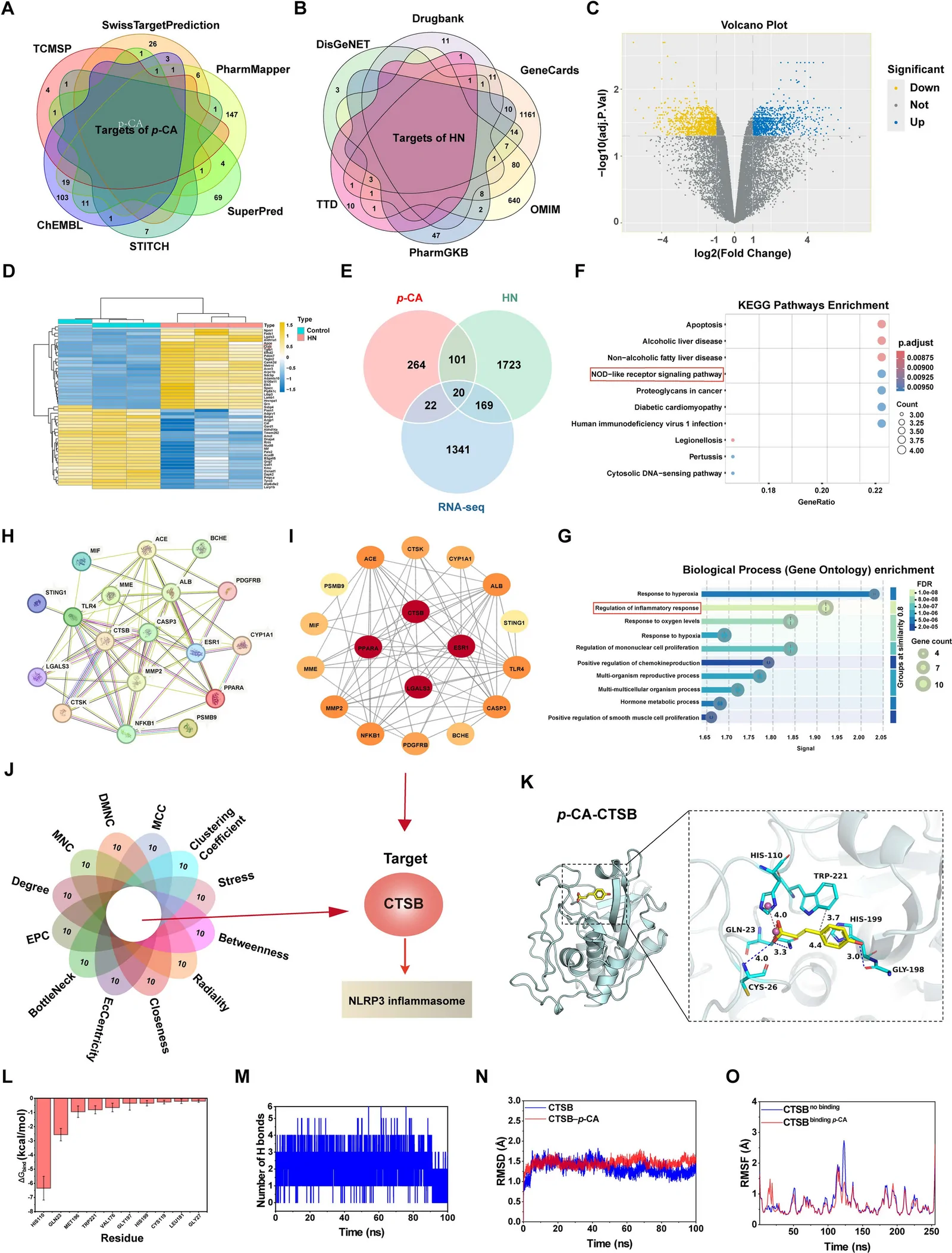
Identification of CTSB as a functional target of *p*-CA. **A** Potential bioavailable targets of *p*-CA were predicted by TCMSP, SuperPRED, SwissTargetPrediction, PharmMapper, ChemBL, and STITCH databases. **B** Genes associated with HN were summarized in DisGeNET, DrugBank, GeneCards, PharmGKB, TTD, and OMIM databases. **C** Volcano plots depicting renal differentially expressed genes in HN rats. **D** Heatmap visualizing the expression profiles of the top 50 most significantly differentially expressed genes in kidneys from HN rats versus control rats. **E** Venn plot showing intersecting targets among *p*-CA, HN, and RNA-seq analyses. **F** Dot plot showing overlapping genes from KEGG enrichment analysis. **G** Overlapping genes from GO enrichment analysis. **H** Protein–protein interaction network generated using STRING for overlapping genes. **I** Network components with the highest scores identified using Cytoscape’s MCODE algorithm. **J** The top 10 genes from cytoHubba’s DMNC, MNC, MCC, EPC, Degree, Eccentricity, BottleNeck, Clustering Coefficient, Radiality, Closeness, Stress, and Betweenness, showing intersection. **K** Molecular docking of CTSB and *p*-CA. Intermolecular interactions, including hydrogen bonds (blue), salt bridges (magenta), and hydrophobic interactions (gray), are indicated by dashed lines, with the corresponding interaction distances explicitly labeled for the critical residues. **L** Per-residue energy decomposition analysis highlighting the top amino acids contributing to the binding free energy of the CTSB-p-CA complex. **M** Dynamic changes in hydrogen bond formation between *p*-CA and CTSB during molecular dynamics simulation (MDS). (N) RMSD profile of *p*-CA in complex with CTSB during molecular dynamics simulation. **O** RMSF profiles of the residues

To elucidate the mechanism by which *p*-CA blocks the pro-fibrotic and pro-inflammatory NLRP3 cascade, we employed a combination of bioinformatics approaches as an exploratory, hypothesis-generating strategy to predict and prioritize potential *p*-CA targets relevant to HN. First, the potential targets of *p*-CA were predicted using TCMSP, SuperPRED, SwissTargetPrediction, PharmMapper, ChemBL, and STITCH databases based on the chemical structure of *p*-CA, yielding 407 targets (Fig. 5A; Additional file 1: Table S5). Concurrently, genes associated with HN were compiled using DisGeNET, DrugBank, GeneCards, PharmGKB, TTD, and OMIM databases, yielding 2013 potential gene targets related to HN (Fig. 5B; Additional file 1: Table S6). To complement these predictions, RNA-seq transcriptomic analysis was performed on kidney tissues of rats to compare genome-wide mRNA expression profiles. The analysis revealed 894 upregulated and 973 downregulated genes in the HN group compared to the control group (Fig. 5C). A heatmap was generated to visualize the expression profiles of the top 50 most significantly differentially expressed genes (Fig. 5D). Intersection of these datasets highlighted 20 overlapping genes among *p*-CA, HN, and RNA-seq profiles as candidate therapeutic targets (Fig. 5E; Additional file 1: Table S7). Pathway enrichment analysis (Fig. 5F) highlighted significant enrichment of the NOD-like receptor pathway among the candidate targets, with NLRP3 serving as a central component (Paik et al. 2021). Consistent with our in vitro and in vivo findings that *p*-CA suppresses this cascade, GO analysis showed that these targets were highly enriched in biological processes, including inflammatory responses and oxidative stress (Fig. 5G).

To further prioritize candidate hub genes, we employed the STRING database for protein–protein interaction analysis by setting a confidence score cutoff of > 0.4 and removing disconnected nodes (Fig. 5H). We then identified hub genes within this network using the 12 algorithms of the CytoHubba plug-in and the MCODE plug-in (Fig. 5I-J). A literature review revealed that CTSB, a lysosomal cysteine protease, is a known regulator of NLRP3 inflammasome activation (Campden and Zhang 2019). CTSB dysregulation has been linked to various inflammatory diseases, metabolic disorders, cancer, and kidney diseases (Cocchiaro et al. 2017). Consistent with our findings, CTSB expression was upregulated at the transcriptional level in the kidney tissues of HN mice from GEO datasets (GSE262687 and GSE190205) (Additional file 1: Figure S5A-B).

Subsequently, we performed molecular docking to evaluate the potential for direct binding between *p*-CA and CTSB (Fig. 5K). The analysis revealed that* p*-CA forms hydrogen bonds with Cys26, Gln23, and Gly198, along with a salt bridge with His110 within the active pocket of CTSB. In addition, hydrophobic interactions with His199 and Trp221 further stabilized the complex. Notably, CTSB contains a conserved catalytic dyad composed of Cys26 and His199, together with a unique occluding loop that includes His110 and regulates substrate recognition and enzymatic activity (Pavlova et al. 2000; Schmitz et al. 2019). In this structural context, the docking results support a model in which *p*-CA may inhibit CTSB enzymatic activity by engaging residues within the catalytic center and occluding-loop region, thereby potentially affecting both catalytic-site accessibility and occluding-loop flexibility.

We next explored the atomistic binding dynamics between *p*-CA and CTSB via molecular dynamics simulations (MDS). Using the MM/GBSA approach, the binding free energy was calculated as −15.38 ± 1.83 kcal/mol (Additional file 1: Table S8), serving as a qualitative indicator of complex stability despite the inherent limitations of implicit solvent models. Energy decomposition analysis (Fig. 5L) identified His110 as the top binding contributor. Notably, this interaction was predominantly driven by non-electrostatic forces, while the observed positive electrostatic term likely reflects a high desolvation penalty characteristic of this modeling environment. Hydrogen bond analysis revealed that *p*-CA and CTSB formed an average of 2 hydrogen bonds (Range: 0–6), further stabilizing the complex (Fig. 5M). Root-mean-square deviation (RMSD) analysis demonstrated that the system equilibrated after initial fluctuations, with no abrupt deviations, indicating stable binding of *p*-CA to CTSB throughout the simulation (Fig. 5N). Furthermore, root mean square fluctuation (RMSF) analysis showed that CTSB bound to *p*-CA exhibited lower flexibility across most regions compared to unliganded CTSB, suggesting that *p*-CA suppresses CTSB flexibility and enhances binding stability (Fig. 5O). Collectively, these computational analyses support a stable CTSB-*p*-CA binding model and provide a structural rationale for subsequent biophysical validation.

### *p*-CA inhibits CTSB activity by engaging its active-site/occluding-loop region

**Fig. 6:**
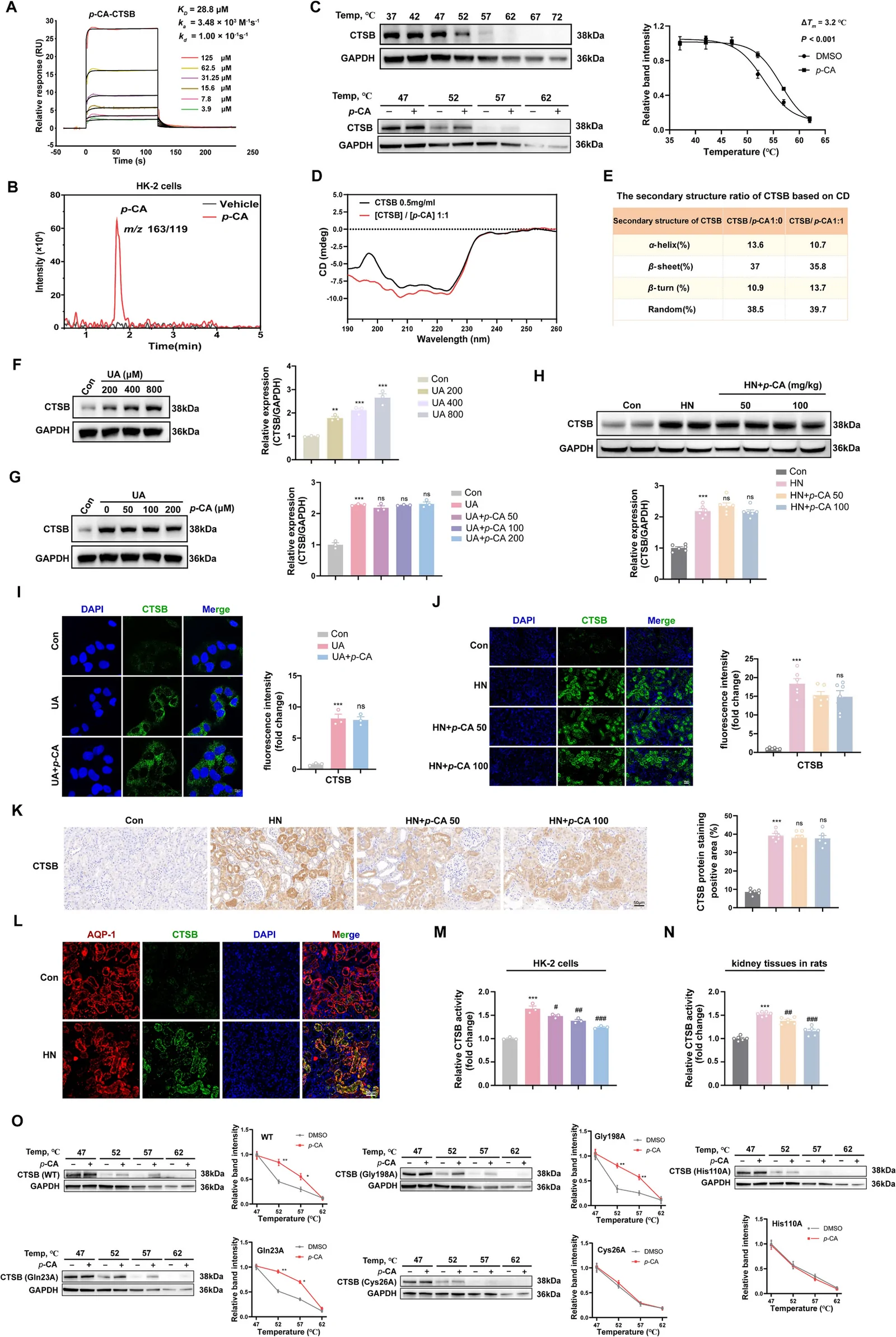
*p*-CA inhibits CTSB activity by engaging its active-site/occluding-loop region. **A** Surface plasmon resonance (SPR) analysis showing the binding affinity between *p*-CA and CTSB. The sensorgram was globally fitted to a 1:1 Langmuir binding model to determine the association rate constant (*k*_*a*_), dissociation rate constant (*k*_*d*_), and equilibrium dissociation constant (*K*_*D*_). **B** High-performance liquid chromatography-tandem mass spectrometry (HPLC–MS/MS) analysis verifying the intracellular entry of *p*-CA in HK-2 cells. **C** Cellular thermal shift assay (CETSA) demonstrating the intracellular target engagement of *p*-CA. Boltzmann sigmoidal melt curves were used to quantify the relative soluble CTSB signal across a thermal gradient in vehicle- (DMSO) or *p*-CA-treated (200 μM) HK-2 cells (Δ*T*_*m*_ of 3.2 °C, *P* < 0.001, determined using the extra sum-of-squares F test). **D** Circular dichroism spectroscopy analysis of *p*-CA-mediated recombinant CTSB conformational changes. (E) Proportions of secondary structure of CTSB with or without *p*-CA by Circular Dichroism-Secondary Structure Estimation software. **F** Western blot analysis of CTSB expression in HK-2 cells treated with a dose gradient of UA (200–800 μM). **G**, **H** Representative Western blots and corresponding quantification of CTSB levels in (**G**) UA-stimulated HK-2 cells treated with *p*-CA (50–200 μM) and (**H**) renal tissues from the indicated rat groups (*n* = 6). **I**, **J** Immunofluorescence images and quantification of CTSB (green) in (**I**) HK-2 cells (Scale bar = 15 μm) and (**J**) rat renal tissues (Scale bar = 50 μm; *n* = 6). **K** Representative Immunohistochemical images of CTSB and quantification of CTSB-positive area (*n* = 6). Scale bar = 50 μm. (**L**) Immunofluorescence co-staining of CTSB and AQP-1 in renal tissues. Scale bar = 50 μm. **M**, **N** Relative CTSB enzymatic activity measured by fluorometric assay in (**M**) HK-2 cells and (**N**) rat kidney tissues (*n* = 6). (**O**) CETSA evaluating *p*-CA-associated target engagement with wild-type (WT) and point-mutant CTSB variants (Gly198A, Gln23A, Cys26A, His110A). For in vitro assays (C, F, G, I, M, O), *n* = 3 independent experiments. All data are expressed as means ± SEM. Statistical significance was determined using one-way ANOVA followed by Tukey’s multiple comparisons test (for F–N) and two-way ANOVA followed by Bonferroni’s multiple comparisons test (for O). A formal test for linear trend was confirmed using simple linear regression across the concentration gradients (all *P*_*trend*_ < 0.001). ^*^P < 0.05, ^**^P < 0.01, and ^***^P < 0.001 *vs.* control group; ns, not significant; ^#^*P* < 0.05, ^##^*P* < 0.01, and ^###^*P* < 0.001 *vs.* the UA or HN group

To further investigate the interaction between *p*-CA and CTSB and its biological effects, we employed SPR analysis. Global fitting of the sensorgrams to a 1:1 Langmuir binding model showed dose-dependent binding with an acceptable fit (χ^2^ = 0.039). This fit is consistent with a single-site engagement model, although complex allosteric or multimode interactions at higher local concentrations cannot be definitively excluded. The kinetic parameters revealed a fast-on/fast-off profile typical of small-molecule interactions, yielding a steady-state equilibrium dissociation constant (*K*_*D*_) of 28.8 μM (Fig. 6A). Subsequent HPLC–MS/MS analysis verified the intracellular accumulation of *p*-CA in HK-2 cells (Fig. 6B). To assess intracellular CTSB target engagement, we performed a cellular thermal shift assay (CETSA). Nonlinear regression analysis using a Boltzmann sigmoidal model revealed that *p*-CA increased the apparent melting temperature (Δ*T*_*m*_) of CTSB by 3.2 °C (*P* < 0.001), supporting intracellular CTSB target engagement by *p*-CA (Fig. 6C). Since ligand binding can induce conformational changes that modulate protein activity (Kenakin and Miller 2010), and given the unique structural features of the CTSB active site—specifically its catalytic dyad and occluding loop—we hypothesized that *p*-CA could induce conformational changes in CTSB. To test this hypothesis, we employed circular dichroism (CD) spectroscopy, a powerful tool for studying the secondary structures and conformational changes of biological macromolecules and molecular interactions (Greenfield 2006). CD spectra of CTSB were recorded between 190 and 260 nm with or without equimolar *p*-CA (*p*-CA/CTSB 1:1) (Fig. 6D). In the presence of *p*-CA, the circular dichroism spectra of CTSB exhibited notable changes in the 190–225 nm range. Using Circular Dichroism-Secondary Structure Estimation software, we quantified these changes. The native CTSB and the *p*-CA-CTSB complex were primarily composed of *β*-sheets and random coils (Fig. 6E). However, in the presence of *p*-CA, the proportions of *β*-sheets and *α*-helices decreased, while the proportions of random coils and *β*-turns increased. Hence, these findings indicate that *p*-CA induces conformational changes in CTSB.

We next examined whether *p*-CA affects CTSB expression or enzymatic activity. Western blot analysis demonstrated upregulation of CTSB expression in UA-treated HK-2 cells and human primary PTECs (Fig. 6F; Additional file 1: Figure S6A); however, *p*-CA did not alter UA-induced CTSB expression (Fig. 6G; Additional file 1: Figure S6A). Similarly, UA-stimulated HK-2 cells showed elevated CTSB mRNA levels, and this upregulation was not attenuated by *p*-CA treatment (Additional file 1: Figure S6B). In line with in vitro data, *p*-CA administration did not significantly alter the increased CTSB levels in the renal tissues of HN rats (Fig. 6H). Consistent findings were observed during immunofluorescence analysis in HK-2 cells (Fig. 6I) and renal tissues (Fig. 6J) from HN rats. Furthermore, immunohistochemistry provided validation of these findings (Fig. 6K). To identify CTSB-expressing cell types in HN kidneys, co-immunofluorescence staining of CTSB was performed with the proximal tubule marker aquaporin-1 (AQP-1). CTSB was observed to predominantly co-localize with AQP-1 (Fig. 6L), a finding supported by immunohistochemistry on sequential tissue sections (Additional file 1: Figure S6C). Single-cell RNA-seq data analysis from the Kidney Precision Medicine Project (KPMP) further established that CTSB-positive proximal renal tubular epithelial cells in CKD patients exhibited trends similar to those in HN rats (Additional file 1: Figure S6D-E). Collectively, these results demonstrated that *p*-CA did not regulate CTSB expression at the mRNA or protein level, prompting us to explore its effects on CTSB enzymatic activity. Based on the CTSB substrate sequence RR conjugated with AFC (7-amino-4-trifluoromethylcoumarin), CTSB activity was quantified by a fluorescence-based activity test kit (Xu et al. 2023). As illustrated in Fig. 6M-N, CTSB enzymatic activity was significantly enhanced in UA-stimulated HK-2 cells and renal tissues from HN rats. Importantly, treatment with *p*-CA significantly and dose-dependently inhibited this aberrant CTSB activity. To evaluate non-specific cross-reactivity with parallel lysosomal cysteine proteases present in the renal microenvironment, the selectivity of* p*-CA was evaluated against recombinant human CTSL and CTSS—the closest structural homologs of CTSB sharing the conserved catalytic dyad. Cell-free fluorometric inhibitor assays revealed that *p*-CA dose-dependently inhibited CTSB with an IC50 of 32.44 μM, whereas it exhibited no apparent inhibitory activity against CTSL and CTSS (IC50 > 500 μM; Additional file 1: Figure S6F), supporting relative selectivity among these tested cathepsin paralogs.

Finally, to explore the putative binding interface, we performed CETSA using point-mutant CTSB variants. It is well-established that the CTSB active site comprises a conserved catalytic dyad (Cys26 and His199) and a unique occluding loop structure containing His110 (Pavlova et al. 2000; Schmitz et al. 2019), which together regulate substrate access and enzyme catalysis. Guided by this structural basis and our docking predictions, we evaluated specific mutations. *p*-CA increased the thermal stability of overexpressed wild-type (WT) CTSB. This thermal protection was reduced in the Cys26A and His110A mutants, whereas Gln23A and Gly198A retained detectable responsiveness similar to WT CTSB (Fig. 6O). These results are consistent with involvement of the Cys26/His110 region in *p*-CA-associated CTSB target engagement in cells. Input Western blot controls showed broadly comparable basal expression of WT and mutant CTSB constructs at 37 °C (Additional file 1: Figure S6G), although these data do not fully establish folding integrity. Collectively, these data support *p*-CA engagement with the CTSB active-site/occluding-loop region and inhibition of CTSB enzymatic activity without altering CTSB expression.

### Targeting CTSB with *p*-CA rescues WWP1-dependent ubiquitination and degradation of NLRP3

**Fig. 7:**
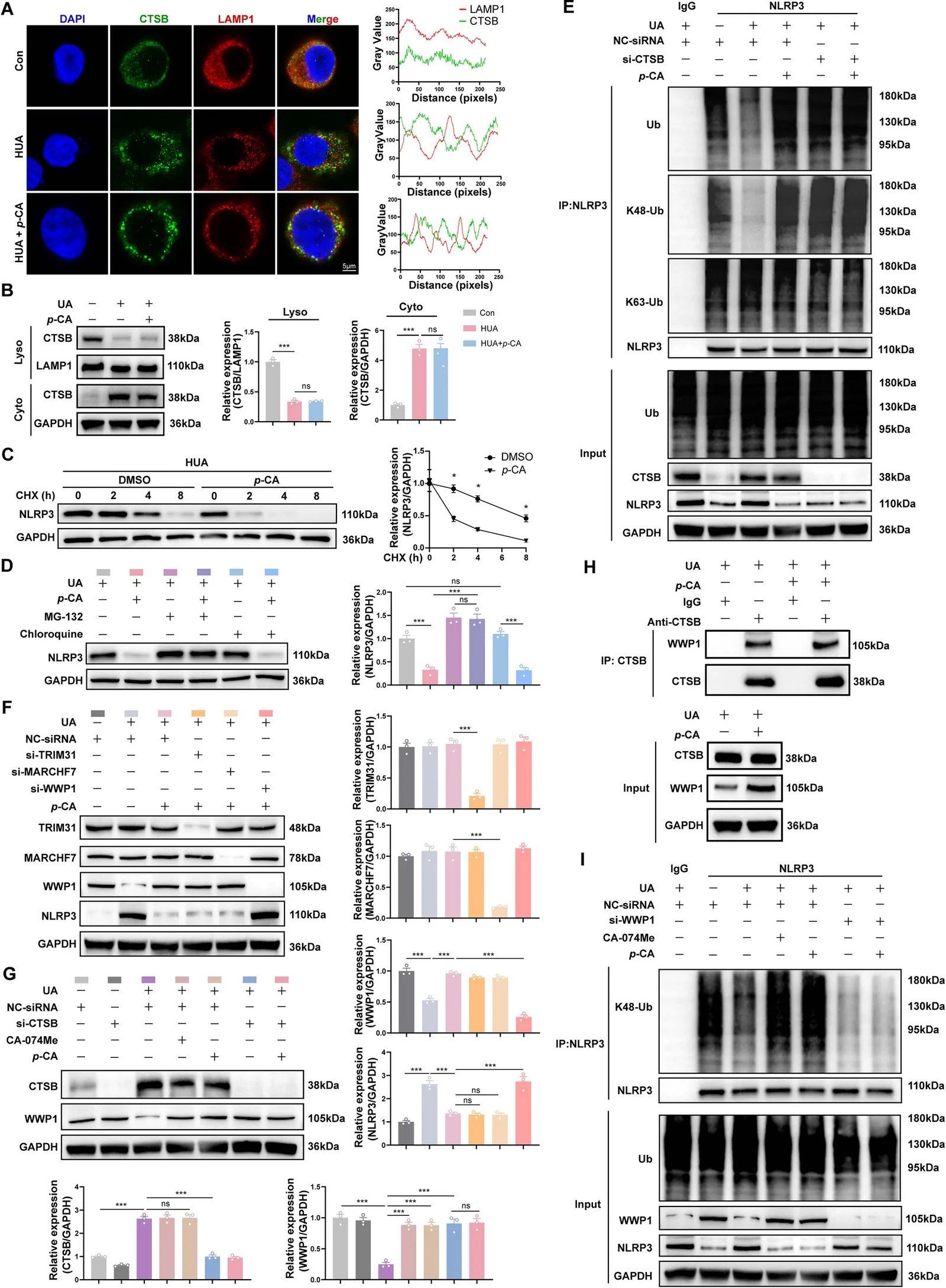
Targeting CTSB with *p*-CA rescues WWP1-dependent ubiquitination and degradation of NLRP3. **A** Representative immunofluorescence images of CTSB (green) and the lysosomal marker LAMP1 (red). The line profile plots (right) indicate the degree of colocalization. Scale bar = 5 μm. **B** Western blot analysis of CTSB distribution in lysosomal (Lyso) and cytosolic (Cyto) fractions isolated from HK-2 cells. LAMP1 and GAPDH served as markers for lysosomes and cytosol, respectively (reciprocal purity controls are presented in Additional file 1: Figure S7B). **C** Cycloheximide (CHX) chase assay evaluating the half-life of NLRP3 protein in UA-stimulated HK-2 cells treated with DMSO or *p*-CA. **D** Western blot analysis and corresponding quantification of NLRP3 in UA-stimulated HK-2 cells co-treated with *p*-CA, the proteasome inhibitor MG-132, or the lysosome inhibitor chloroquine (CQ). **E** Co-immunoprecipitation (Co-IP) assay assessing the specific ubiquitination status of NLRP3. Lysates from HK-2 cells with indicated treatments were immunoprecipitated with an anti-NLRP3 antibody and immunoblotted for pan-ubiquitin (Ub), K48-linked ubiquitin (K48-Ub), K63-linked ubiquitin (K63-Ub), and NLRP3. **F** Representative Western blot analysis of specific E3 ubiquitin ligases (TRIM31, MARCHF7, WWP1) and NLRP3 in HK-2 cells following UA stimulation, specific siRNA knockdowns, and *p*-CA treatment. **G** Representative Western blot analysis revealing the modulatory effects of CTSB knockdown (si-CTSB), CTSB inhibition (CA-074Me), and *p*-CA on WWP1 and NLRP3 protein abundance. **H** Co-IP assay verifying the physical interaction between leaked CTSB and WWP1 in the presence or absence of *p*-CA. **I** Co-IP assay determining the dependence of restored K48-linked ubiquitination of NLRP3 on WWP1, following CTSB inhibition (CA-074Me) or *p*-CA treatment. Lysates from HK-2 cells were immunoprecipitated with anti-NLRP3 and immunoblotted for K48-Ub. For all in vitro assays, n = 3 independent experiments. All data are expressed as means ± SEM. Statistical significance was determined using one-way ANOVA followed by Tukey’s multiple comparisons test (for B, D, F, G) and two-way ANOVA followed by Bonferroni’s multiple comparisons test (for C). ns, not significant. In panel C, ^*^P < 0.05 indicates a significant difference between the DMSO and *p*-CA groups. In the bar graphs, statistical differences between the specifically indicated groups are denoted by connecting lines (^***^P < 0.001)

Lysosomal membrane permeabilization (LMP) is a critical upstream event facilitating the cytosolic release of lysosomal proteases (Wang et al. 2018). Using Acridine Orange (AO) staining, we observed a significant red-to-green fluorescence shift in UA-stimulated HK-2 cells, indicative of severe lysosomal rupture (Additional file 1: Figure S7A). Subcellular fractionation (validated by reciprocal purity controls in Additional file 1: Figure S7B) and immunofluorescence co-staining with the lysosomal marker LAMP1 further confirmed that UA induced a marked translocation of CTSB from intact lysosomes into the cytosol (Fig. 7A-B). Notably, *p*-CA treatment did not restore lysosomal acidity or prevent the cytosolic accumulation of leaked CTSB. These data suggest that *p*-CA functions primarily as a post-lysosomal interceptor, acting downstream of lysosomal rupture to inhibit cytosolic CTSB rather than repairing the lysosomal membrane.

Having obtained evidence that *p*-CA engages CTSB and suppresses its enzymatic activity, we sought to elucidate the precise mechanism by which this interaction mediates the marked reduction in NLRP3 protein levels. To first exclude upstream transcriptional regulation, we evaluated NLRP3 mRNA levels. While UA stimulation significantly upregulated NLRP3 transcription, indicating canonical NF-κB priming, neither *p*-CA nor the CTSB inhibitor CA-074Me altered this transcriptional induction (Additional file 1: Figure S7C). This supports the interpretation that CTSB antagonism mainly regulates NLRP3 at the post-translational level in this UA-induced model. To further determine whether *p*-CA governs NLRP3 protein stability, cycloheximide (CHX) chase assays were performed. The results revealed that *p*-CA treatment significantly accelerated the degradation rate of endogenous NLRP3 proteins in UA-stimulated cells, substantially shortening its half-life (Fig. 7C). To further elucidate the specific degradative pathway of NLRP3, we employed pharmacological inhibitors. Given that UA-induced LMP severely compromises endogenous lysosomal clearance capacity, the addition of the lysosome-autophagy inhibitor chloroquine (CQ) predictably had no significant effect on NLRP3 accumulation. However, the accelerated degradation of NLRP3 by *p*-CA was substantially reversed by the proteasome inhibitor MG-132 (Fig. 7D), supporting proteasomal degradation as a major clearance mechanism for NLRP3 in this context.

Ubiquitination is increasingly recognized as a critical post-translational modification governing NLRP3 inflammasome activation (Beesetti 2025). The linkage type of the polyubiquitin chain dictates the fate of NLRP3: Lys48 (K48)-linked ubiquitination canonically drives its proteasomal degradation, whereas Lys63 (K63)-linkage ubiquitination can direct NLRP3 toward autophagic clearance or signaling modulation. To evaluate the specific ubiquitination status of NLRP3, we performed co-immunoprecipitation assays in the presence of MG-132. In control cells, NLRP3 exhibited a basal level of K48-linked polyubiquitination. UA stimulation markedly diminished this K48-ubiquitination, but had no notable effect on K63-linked ubiquitination, indicating a specific blockade of the degradative signal. Notably, *p*-CA treatment effectively restored the K48-linked polyubiquitination of NLRP3 (Fig. 7E). Subsequent densitometric quantification confirmed that K63-linked ubiquitination levels remained statistically unchanged across all experimental conditions, including *p*-CA treatment or CTSB silencing (Additional file 1: Figure S7D). Furthermore, CTSB silencing (si-CTSB) replicated the restorative effect of *p*-CA on K48-linked ubiquitination of NLRP3, and combining *p*-CA with si-CTSB produced no clear additive effect. These findings are consistent with CTSB pathway dependence in *p*-CA-mediated restoration of NLRP3 K48-linked ubiquitination.

**Fig. 8:**
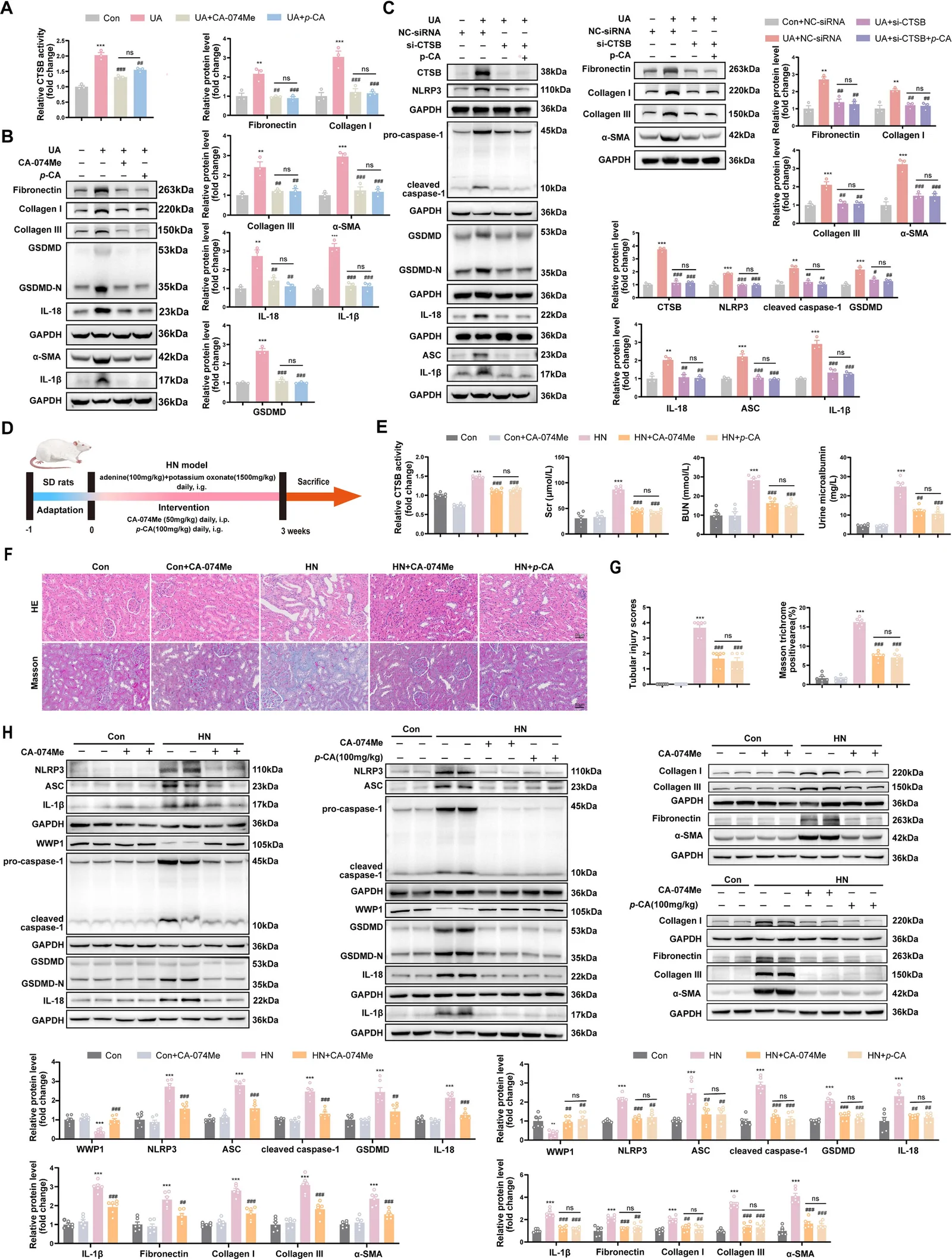
The anti-inflammatory and anti-fibrotic effects of *p*-CA are dependent on CTSB blockade. **A** CTSB activity measured using the CTSB Activity Assay Kit (fluorometric) in HK-2 cells. **B** Western blot analysis and corresponding quantification of NLRP3 inflammasome components and fibrotic markers in UA-stimulated HK-2 cells treated with the CTSB inhibitor CA-074Me or *p*-CA. **C** Western blot analysis evaluating the modulatory effects of *p*-CA on the NLRP3 cascade and fibrosis in UA-stimulated HK-2 cells following CTSB knockdown. **D** Schematic representation of the in vivo experimental design for the CTSB inhibitor CA-074Me or *p*-CA intervention in HN rats. **E** Relative CTSB activity in kidney tissues, along with serum creatinine (Scr), blood urea nitrogen (BUN), and urine microalbumin levels across different rat groups (*n* = 6). **F** Representative images of Hematoxylin and eosin (H&E) and Masson's trichrome staining of rat kidney sections. Scale bar = 50 μm. **G** Quantification of tubular injury scores and Masson's trichrome-positive area (*n* = 6). **H** Representative Western blots and quantitative analysis of WWP1, the NLRP3 inflammatory pathway and fibrotic markers in kidney tissues from the indicated rat groups (*n* = 6). For A–C, *n* = 3 independent experiments. All data are expressed as means ± SEM. Statistical significance was determined using one-way ANOVA followed by Tukey’s multiple comparisons test (for all panels). ns, not significant; ^*^P < 0.05, ^**^P < 0.01, and ^***^P < 0.001 *vs*. control (or Con + NC-siRNA) group; ^#^*P* < 0.05, ^##^*P* < 0.01, and ^###^*P* < 0.001 *vs*. UA (or UA + NC-siRNA or HN) group

Given that K48-linked polyubiquitination is orchestrated by specific E3 ligases, we performed an siRNA-based screen of known NLRP3-targeting candidates (TRIM31, MARCHF7, and WWP1) (Gao et al. 2024; Song et al. 2016; Zhang et al. 2022). Immunoblot analysis revealed that UA stimulation specifically induced the downregulation of WWP1, whereas TRIM31 and MARCHF7 protein levels remained largely unaffected. Notably, *p*-CA treatment effectively rescued WWP1 protein abundance. Moreover, only the knockdown of WWP1, but not TRIM31 or MARCHF7, abolished the suppressive effect of *p*-CA on NLRP3 accumulation (Fig. 7F), identifying WWP1 as a critical E3 ligase in this process. To elucidate this regulatory axis, we investigated whether CTSB, a *p*-CA-engaged target, regulates WWP1 stability. Immunoblot analysis demonstrated that UA stimulation resulted in a marked decrease in WWP1 protein abundance, which was effectively reversed by the CTSB inhibitor CA-074Me, CTSB knockdown, or *p*-CA treatment (Fig. 7G). Importantly, quantitative RT-PCR analysis revealed no significant differences in WWP1 mRNA levels across these corresponding groups (Additional file 1: Figure S7E), suggesting post-translational regulation. As will be detailed in the subsequent in vivo studies (Fig. 8H), this preservation of WWP1 protein abundance by *p*-CA and CA-074Me was also observed in kidney tissues from HN rats. To determine whether CTSB associates with WWP1, Co-IP assays were performed (Fig. 7H). Under UA stimulation, Co-IP detected an interaction between leaked CTSB and WWP1. Notably, while *p*-CA preserved WWP1 protein abundance, it did not disrupt the CTSB-WWP1 association detected by Co-IP. Thus, the persistence of the Co-IP-detected CTSB-WWP1 association should not be interpreted as inconsistent with WWP1 preservation by *p*-CA, because Co-IP reports protein association rather than CTSB catalytic activity or substrate turnover. These findings support an operational model in which CTSB enzymatic activity contributes to post-translational WWP1 destabilization, whereas *p*-CA suppresses CTSB activity and preserves WWP1 abundance without measurably disrupting the CTSB-WWP1 complex under our Co-IP conditions. However, these data do not define whether WWP1 is directly cleaved by CTSB or destabilized through an intermediate mechanism.

Finally, to establish the functional requirement of WWP1 for *p*-CA-restored ubiquitination, we performed further Co-IP analyses. Consistent with the protein expression data, both the CTSB inhibitor CA-074Me and *p*-CA restored the K48-linked polyubiquitination on NLRP3. Crucially, siRNA-mediated silencing of WWP1 substantially abolished the capacity of *p*-CA to restore this K48-ubiquitin modification, resulting in subsequent NLRP3 accumulation (Fig. 7I). Collectively, these results support a model in which *p*-CA preserves WWP1 protein abundance by limiting CTSB activity-associated loss of WWP1 abundance/stability, thereby maintaining WWP1-dependent K48-linked ubiquitination and proteasomal degradation of NLRP3.

### The anti-inflammatory and anti-fibrotic effects of *p*-CA are dependent on CTSB blockade

To assess whether the protective effects of *p*-CA are CTSB-pathway dependent, we utilized CA-074Me, a well-known CTSB inhibitor (Lutgens et al. 2007). In UA-stimulated HK-2 cells, 15 μM CA-074Me markedly inhibited CTSB activity and downregulated proteins associated with inflammation, pyroptosis, and fibrosis, showing a protective pattern similar to that of 200 μM *p*-CA (Fig. 8A-B). To further assess this dependency, we performed genetic silencing of CTSB using siRNA. Knockdown of CTSB significantly reduced the expression of NLRP3 inflammasome components (ASC, NLRP3, GSDMD-N, cleaved caspase-1, IL-1β, IL-18) and fibrotic markers (Collagen I/III, Fibronectin, α-SMA). Crucially, in CTSB-depleted cells, *p*-CA treatment did not exert additional suppressive effects on these signaling pathways (Fig. 8C). This lack of clear additional efficacy after CTSB silencing is consistent with substantial CTSB pathway dependence of *p*-CA in suppressing the NLRP3 cascade and fibrotic responses.

We further validated these findings in vivo by comparing the pharmacological effects of *p*-CA (100 mg/kg) and the CTSB inhibitor CA-074Me (50 mg/kg), which served as a positive control, on NLRP3 inflammasome-mediated inflammation and fibrosis in HN rats (Fig. 8D). Consistent with our in vitro data, CA-074Me treatment significantly attenuated CTSB activity, improved renal function (Scr, BUN, urinary microalbumin), and alleviated tubular injury and fibrosis, as confirmed by Masson’s and H&E staining. Notably, *p*-CA exhibited a broadly similar protective pattern to CA-074Me in suppressing CTSB activity and ameliorating histopathological injury (Fig. 8E-G). Mechanistically, Western blot analyses of renal tissues revealed that both compounds effectively restored WWP1 protein expression, alongside a parallel and significant downregulation of the NLRP3 inflammasome axis and key fibrotic markers (Fig. 8H). However, a distinct divergence was observed in their short-term tolerability. While CA-074Me-treated rats exhibited signs of systemic toxicity, including reduced weight gain and liver dysfunction, *p*-CA treatment induced no overt signs of systemic or hepatic toxicity within the 3-week experimental window (Additional file 1: Figure S8A-B). Collectively, these findings support that *p*-CA attenuates inflammation and fibrosis, at least in substantial part, through the CTSB-WWP1-NLRP3 pathway in this model.

### Clinical relevance of CTSB expression in hyperuricemia-associated CKD patients

**Fig. 9:**
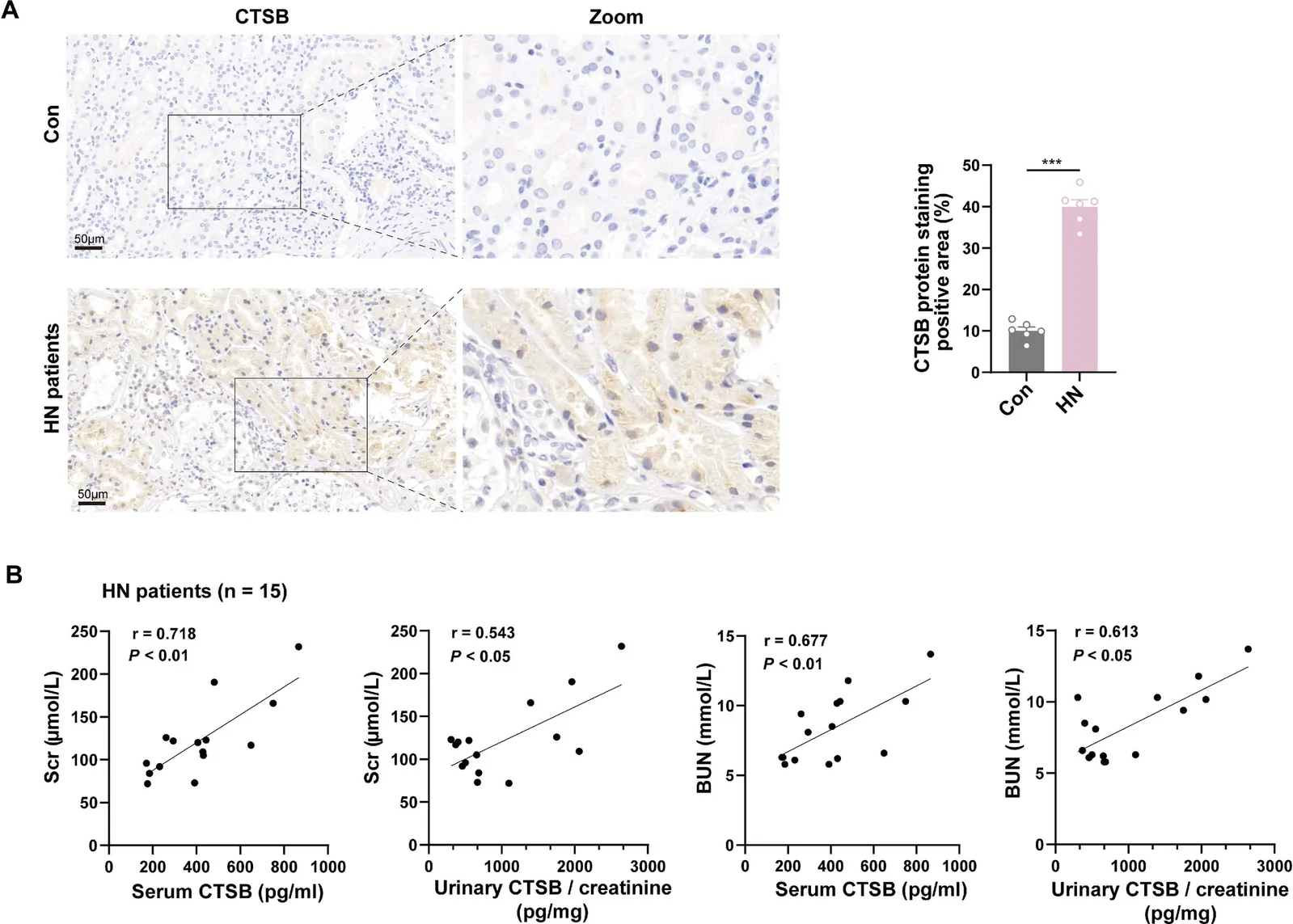
Clinical relevance of CTSB expression in the hyperuricemia-associated CKD cohort. **A** Representative immunohistochemical staining of CTSB in renal tissues from healthy controls (Con) and patients with hyperuricemia-associated CKD, with the histogram on the right showing the quantification of the positive area (*n* = 6). Scale bar = 50 μm. **B** Spearman’s correlation analysis showing the relationships between renal function markers (Scr, BUN) and CTSB levels (Serum/Urinary) in the hyperuricemia-associated CKD cohort (*n* = 15). Statistical significance was determined using an unpaired Student's t-test (for A) and Spearman's correlation analysis (for B). All data are expressed as means ± SEM. ^***^P < 0.001 *vs*. control group

To assess the clinical relevance of the CTSB-associated pathological cascade observed in our experimental models, we investigated CTSB expression profiles in patients from our hyperuricemia-associated CKD cohort. Immunohistochemical analysis of human renal biopsies revealed increased CTSB expression, predominantly localized to renal tubules and interstitium in these patients compared to normal tissues (Fig. 9A). Furthermore, in our clinical cohort (n = 15), both serum and urinary CTSB levels were positively correlated with indicators of renal dysfunction (Scr and BUN) (Fig. 9B). These clinical data support the relevance of CTSB dysregulation in the hyperuricemia-associated CKD cohort and are consistent with our experimental findings.

## Discussion

Hyperuricemic nephropathy remains a complex pathological process centered on the persistent urate-induced activation of the NLRP3 inflammasome and subsequent tubulointerstitial fibrosis. Despite advances in understanding its pathogenesis, effective therapies that directly target the intrarenal inflammatory microenvironment without solely relying on systemic urate reduction are still lacking*.* In this study, through a targeted phenotypic screen, we identified the natural phenolic compound *p*-CA as a potent pharmacological candidate in HN. We provide systematic evidence supporting the notion that *p*-CA suppresses CTSB enzymatic activity by engaging the CTSB active-site/occluding-loop region involving Cys26 and His110. By acting as a post-lysosomal interceptor, *p*-CA prevents CTSB activity-associated reduction of the E3 ubiquitin ligase WWP1 protein abundance. This intervention rescues the WWP1-dependent K48-linked polyubiquitination and subsequent proteasomal clearance of NLRP3, thereby attenuating NLRP3 inflammasome activation, pyroptosis, inflammatory cascades, and tubulointerstitial fibrosis.

Central to our findings is the identification of CTSB as a functionally relevant pharmacological target of *p*-CA in the UA-induced tubular injury model. While our multidimensional network pharmacology screening served primarily as an exploratory tool for initial target prioritization, our mechanistic conclusions are supported by the subsequent target-engagement and biochemical validations. While previous studies have described the cytoprotective potential of *p*-CA in obstructive and heavy metal-induced nephropathies via generic antioxidant and immunomodulatory pathways (Navaneethan and Rasool 2014; Yao et al. 2025), or attributed its inhibition of lysosomal enzymes to generic membrane-stabilizing effects mediated by ROS scavenging in myocardial infarction models (Jyoti and Stanely 2013), our biophysical (SPR, CETSA, and CD spectroscopy) and subcellular analyses reveal a distinct non-canonical mechanism. Under severe hyperuricemic stress, *p*-CA does not prevent lysosomal membrane permeabilization (LMP); rather, it directly inhibits cytosolic CTSB. Using computational hotspot mapping (MM/GBSA), SPR-based biophysical analysis, and cellular target-engagement validation by site-directed CETSA, we identified catalytic residue Cys26 and the occluding-loop residue His110 as key residues associated with *p*-CA-mediated CTSB target engagement in cells. Notably, while static computational docking suggested a relatively modest energetic contribution from Cys26, our site-directed CETSA revealed a marked reduction in *p*-CA-associated thermal protection in the Cys26A mutant. This discrepancy is consistent with a contribution of Cys26 to *p*-CA-mediated CTSB target engagement in the cellular CETSA assay, a functional nuance often underestimated by static in silico models. Furthermore, although our overall computational models predicted a strong theoretical binding energy, our SPR analysis characterized this interaction as a moderate, fast-on/fast-off binding profile typical of small-molecule inhibitors. Moreover, the higher extracellular concentration used for CETSA and cell-based assays, relative to the SPR-derived *K*_*D*_, is consistent with the need to overcome cellular barriers, intracellular competition, and target-occupancy constraints in the complex intracellular milieu. Structurally, the CTSB active site features a unique occluding loop critical for allosteric regulation, which is stabilized by the pivotal His110–Asp22 salt bridge (Pavlova et al. 2000; Schmitz et al. 2019). By engaging His110 and surrounding pocket residues, *p*-CA may perturb this interaction, thereby limiting the conformational flexibility required for enzyme catalysis. Together, these data support *p*-CA as a CTSB-directed inhibitor with preferential activity over the tested renal cathepsin homologs in this pathological context, rather than acting solely through generic transcriptional suppression.

Current studies indicate that the role of CTSB in renal pathology remains complex and context-dependent. While elevated CTSB correlates with age-related renal decline (Wang et al. 2016) and diabetic nephropathy (Musante et al. 2015), other studies suggest decreased activity in polycystic kidney disease (Peintner et al. 2021) and experimental diabetic nephropathy models (Grzebyk et al. 2013; Peres et al. 2013), highlighting a disease-specific functional dichotomy. In the specific context of HN, although a recent experimental study implicated CTSB in its pathogenesis (Hu et al. 2022), its clinical relevance, targeted interventional strategies, and precise downstream effectors remained incompletely defined. To resolve this context-dependent ambiguity and bridge the translational gap, we provided cohort-level clinical evidence showing aberrant CTSB and NLRP3 upregulation in the renal tubules and interstitium of patients from our hyperuricemia-associated CKD cohort. Crucially, both serum and urinary CTSB levels exhibited a positive correlation with renal dysfunction markers (Scr and BUN). By combining genetic knockdown with pharmacological inhibition, we provide evidence supporting CTSB as a pathogenic mediator in HN progression and indicate that the renoprotective efficacy of *p*-CA is, at least in substantial part, CTSB pathway-dependent. This context-dependent role also extends to urate metabolism. Prior work suggested that CTSB inhibition lowers uric acid by downregulating URAT1/GLUT9 (Lin et al. 2025). However, in our HN model, *p*-CA-mediated CTSB inhibition ameliorated kidney pathology without altering systemic urate levels or transporter expression. This uncoupling demonstrates that *p*-CA’s renoprotection stems from dismantling the post-lysosomal inflammatory cascade rather than relying on secondary urate metabolic regulation, providing a rationale for further evaluating *p*-CA as an adjunctive strategy in patients who remain at risk of renal progression despite urate-lowering therapy.

Another key contribution of our study is the identification of the E3 ubiquitin ligase WWP1 as a mechanistic link between cytosolic CTSB activity and NLRP3-driven inflammation. While lysosomal CTSB leakage is widely recognized as a trigger for NLRP3 inflammasome activation (Campden and Zhang 2019), the precise molecular mechanisms bridging CTSB activity and NLRP3 accumulation have remained elusive. We bridge this gap by unveiling a previously unrecognized CTSB-WWP1-NLRP3 degradation axis. Emerging evidence (Zeng et al. 2024; Zhang et al. 2022) has established that WWP1 negatively regulates NLRP3 in models of sepsis and atopic dermatitis by specifically driving its K48-linked polyubiquitination and subsequent clearance via the ubiquitin–proteasome system (UPS). We extend this paradigm by identifying an upstream pathological event in HN: following UA-induced lysosomal membrane permeabilization (LMP), leaked cytosolic CTSB associates with WWP1 and promotes CTSB activity-sensitive loss of WWP1 protein abundance. Biochemically, our Co-IP assays support the formation of CTSB-WWP1-containing complexes in cells. Because lysates were prepared under cold conditions with broad-spectrum protease inhibitors, these assays are expected to minimize post-lysis proteolysis and preserve pre-existing protein associations. However, these data do not establish WWP1 as a direct proteolytic substrate of CTSB, nor do they define the precise molecular step that couples CTSB activity to WWP1 turnover. The persistence of Co-IP-detected CTSB-WWP1 association in the presence of *p*-CA suggests that *p*-CA preserves WWP1 by inhibiting CTSB catalytic activity rather than by dissociating the CTSB-WWP1 complex. Alternative mechanisms remain possible, including direct limited proteolysis of WWP1, CTSB-dependent recruitment or activation of an intermediate proteolytic/degradation pathway, or secondary turnover through proteasomal or autophagy-related mechanisms. Together with the reduced WWP1 protein abundance/stability observed under UA stimulation, these findings support a model in which CTSB activity-sensitive loss of WWP1 protein abundance impairs WWP1-mediated NLRP3 ubiquitination-dependent degradation, thereby facilitating inflammasome activation. To restore this regulatory axis, our pharmacological (MG-132 versus CQ) and genetic analyses support that *p*-CA preserves WWP1-mediated NLRP3 clearance through the K48-linked UPS pathway in HN, thereby attenuating inflammasome activation. By inhibiting CTSB enzymatic activity, *p*-CA functions as a post-lysosomal interceptor that preserves WWP1 protein abundance. Consequently, this intervention supports WWP1-mediated K48-linked ubiquitination and proteasomal degradation of NLRP3 even when lysosomal clearance capacity is impaired. By limiting NLRP3 accumulation, *p*-CA attenuates inflammasome assembly and caspase-1-dependent cytokine maturation/release, thereby reducing downstream inflammatory amplification without measurably suppressing pro-IL-1β priming.

Notably, *p*-CA exhibited a more favorable short-term tolerability compared to the synthetic CTSB inhibitor CA-074Me. While both compounds demonstrated comparable efficacy in inhibiting the NLRP3 cascade, CA-074Me induced signs of systemic and hepatic stress, likely due to off-target inhibition of other essential cathepsins during prolonged exposure—a common bottleneck for synthetic protease inhibitors. In contrast, *p*-CA treatment showed no overt signs of hepatotoxicity within our 3-week observation period. Future long-term studies are required to establish a comprehensive safety profile. Coupled with favorable pharmacokinetic properties—including rapid intestinal absorption, efficient renal excretion, and high bioavailability compared to other phenolic compounds (Abazari et al. 2021)—*p*-CA emerges as a promising pharmacological candidate for HN. Nevertheless, direct in vivo PK/PD validation in the HN model remains necessary before inferring confirmed renal accumulation or target occupancy.

Several limitations warrant consideration. First, although our data support a CTSB-WWP1 association and a CTSB activity-sensitive reduction in WWP1 protein abundance/stability, the precise molecular mechanism underlying CTSB-associated WWP1 destabilization remains unresolved. The current data do not establish WWP1 as a direct CTSB substrate. Future recombinant cleavage assays with active and catalytically inactive CTSB, recombinant WWP1, and cleavage-site mapping will be required to resolve whether WWP1 is directly cleaved by CTSB or destabilized through an intermediate mechanism. Second, clinical translation must account for inherent interspecies differences, notably the presence of uricase in rodents, which alters urate dynamics compared to humans. The adenine and potassium oxonate-induced model also encompasses both urate-driven and adenine crystal-associated tubular injuries, whereas human HN usually develops over decades in the setting of complex metabolic comorbidities. In addition, we did not test canonical LPS-primed inflammasome models; therefore, our mechanistic interpretation should be limited to the context of UA-induced sterile tubular injury and HN-associated renal inflammation. Third, the exact pharmacokinetic profile of *p*-CA in our HN model, including plasma and renal tissue concentrations measured by HPLC–MS/MS, was not directly evaluated. Although previous pharmacokinetic studies suggest rapid renal excretion and possible tubular handling of phenolic acids (Garrait et al. 2006; Lu et al. 2022), these data cannot establish renal *p*-CA exposure or CTSB target occupancy in our experimental model. Formal PK/PD mapping will therefore be essential to determine whether renal *p*-CA concentrations reach the range required for CTSB inhibition and to guide future dose optimization. Fourth, although our findings support CTSB as a functionally relevant target of *p*-CA in the UA-induced tubular injury model, *p*-CA is a phenolic compound with known pleiotropic properties. Because mitochondrial function and apoptosis-specific markers were not directly assessed, parallel mitochondrial or apoptotic regulation cannot be excluded. Fifth, purified WT and mutant CTSB binding assays, such as SPR or ITC using recombinant CTSB variants, and siRNA-resistant WT and catalytically inactive CTSB rescue experiments were not performed; therefore, future studies are required to further validate residue-level binding, thermodynamic interaction parameters, and genetic evidence for on-target specificity. Finally, the broad-spectrum applicability of this CTSB-WWP1 axis in other metabolic or fibrotic kidney diseases characterized by lysosomal stress warrants further investigation.

## Conclusions

In summary, our study supports that *p*-CA ameliorates HN by targeting the CTSB-WWP1-NLRP3 signaling axis. Mechanistically, *p-*CA engages the CTSB active-site/occluding-loop region involving Cys26 and His110, thereby inhibiting CTSB enzymatic activity and limiting CTSB activity-associated reduction in WWP1 protein abundance. By preserving WWP1 protein abundance, *p-*CA restores the K48-linked polyubiquitination and proteasomal clearance of NLRP3, thereby suppressing inflammasome activation. This intervention attenuates downstream inflammation, cellular pyroptosis, and renal fibrosis. These findings support a previously unrecognized pathogenic mechanism contributing to HN, whereby lysosomal damage promotes inflammasome hyperactivation through disrupted ubiquitin-dependent clearance. The precise molecular mechanism linking CTSB activity to WWP1 turnover remains unresolved and requires further biochemical validation. Our findings provide a molecular rationale for evaluating *p*-CA as a prophylactic or early co-treatment strategy against this progressive nephropathy.

## Supplementary Information

Additional file 1.

## Data Availability

The datasets generated during and/or analyzed during the current study are available from the corresponding author upon reasonable request.

## Abbreviations

ALT: Alanine aminotransferase
AO: Acridine orange
AQP-1: Aquaporin-1
AST: Aspartate aminotransferase
α-SMA: α-Smooth muscle actin
BUN: Blood urea nitrogen
CCK-8: Cell counting kit-8
CETSA: Cellular thermal shift assay
CHX: Cycloheximide
CKD: Chronic kidney disease
Co-IP: Co-immunoprecipitation
CQ: Chloroquine
CTSB: Cathepsin B
CTSL: Cathepsin L
CTSS: Cathepsin S
GEO: Gene Expression Omnibus
GSDMD: Gasdermin D
H&E: Hematoxylin and eosin
HK-2: Human proximal tubule cell line
HN: Hyperuricemic nephropathy
HPLC–MS/MS: High performance liquid chromatography-tandem mass spectrometry
IL-18: Interleukin-18
IL-1β: Interleukin-1β
LAMP1: Lysosome-associated membrane protein 1
LMP: Lysosomal membrane permeabilization
MDS: Molecular dynamics simulation
MM/GBSA: Molecular Mechanics/Generalized Born Surface Area
MPO: Myeloperoxidase
NLRP3: NLR family pyrin domain containing 3
PAS: Periodic acid-Schiff
*p*-CA: P-Coumaric acid
PTECs: Human primary proximal tubular epithelial cells
RMSD: Root-mean-square deviation
RMSF: Root mean square fluctuation
Scr: Serum creatinine
siRNA: Small interfering RNA
SPR: Surface plasmon resonance
SUA: Serum uric acid
UA: Uric acid
UMAP: Uniform Manifold Approximation and Projection
UPS: Ubiquitin-proteasome system
WT: Wild-type
WWP1: WW domain-containing E3 ubiquitin protein ligase 1
