# Supplementary material for: Targeting Cathepsin B with p-coumaric acid rescues WWP1-dependent proteasomal degradation of NLRP3 in hyperuricemic nephropathy
Source: Mol Med. 2026 Jun 26;32:143. doi: 10.1186/s10020-026-01544-y (PMC13563950; doi:10.1186/s10020-026-01544-y)
Supplement: Supplementary file 1 [file 10020_2026_1544_MOESM1_ESM.docx]

# Targeting Cathepsin B with *p*-Coumaric acid rescues WWP1-dependent proteasomal degradation of NLRP3 in hyperuricemic nephropathy

## Supplementary Information

The supplementary information includes:

**Figure S1** Validation of UA-induced NLRP3 activation and evaluation of cellular pro-IL-1β levels following *p*-CA treatment

**Figure S2** The effects of *p*-CA on urate transporters, body weight, serum transaminases, and pro-IL-1β levels in HN rats

**Figure S3** Analysis of pyroptosis and inflammation in HN

**Figure S4** The effects of *p*-CA on fibrotic markers in UA-induced human primary PTECs

**Figure S5** Analysis of the differentially expressed genes in HN mice from the GEO database

**Figure S6** Expression profiles of CTSB, evaluation of target selectivity, and validation of mutant stability

**Figure S7** Evaluation of lysosomal membrane permeabilization, NLRP3 and WWP1 mRNA transcription, and K63-linked ubiquitination

**Figure S8** Comparative effects of *p*-CA versus CA-074Me on body weight and serum transaminases *in vivo*

**Table S1** Clinical characteristics of patients from the hyperuricemia-associated CKD cohort

**Table S2** Selected phenolic compounds

**Table S3** Sequences of small interfering RNAs (siRNAs) used in this study

**Table S4** Functional roles and structural mapping of key CTSB residues

**Table S5** Potential bioavailable targets of *p*-CA predicted by TCMSP, SuperPRED, SwissTargetPrediction, PharmMapper, ChemBL and STITCH databases

**Table S6** Genes associated with HN summarized in DisGeNET, DrugBank, GeneCards, PharmGKB, TTD and OMIM database

**Table S7** Common target proteins selected for network pharmacology and RNA-seq

**Table S8** Binding free energies and energy components of CTSB-*p*-CA predicted by MM/GBSA (kcal/mol)

**Figure S1.** **Validation of UA-induced NLRP3 activation and evaluation of cellular pro-IL-1β levels following *p*-CA treatment**. (A) Immunofluorescence analysis of the colocalization between NLRP3 and the proximal tubule marker AQP-1 in kidney sections of HN rats. Scale bar = 50 μm. (B) Representative Western blots of NLRP3, caspase-1, IL-18, Fibronectin, Collagen I, and Collagen III proteins, and quantification of protein levels normalized to GAPDH in HK-2 cells induced by UA at 200-800 μM. (C) Immunoblot analysis and corresponding quantification of total pro-IL-1β protein levels in UA-stimulated HK-2 cells following *p*-CA treatment. n = 3 independent experiments. Data are presented as means ± SEM. Statistical significance was determined using one-way ANOVA followed by Tukey’s multiple comparisons test. A formal test for linear trend was confirmed using simple linear regression across the UA concentration gradient in (B) (*P_trend_* < 0.001). ^*^*P* < 0.05, ^**^*P* < 0.01, and ^***^*P* < 0.001 versus the control group. ns, not significant versus the UA group. DAPI, 4',6-diamidino-2-phenylindole; AQP-1, aquaporin 1.

**Figure S2. The effects of *p*-CA on urate transporters, body weight, serum transaminases, and pro-IL-1β levels in HN rats.** (A) Western blotting and quantitative analysis of URAT1 and GLUT9 in kidney tissues (n = 6). (B) Immunoblot analysis and corresponding quantification of total pro-IL-1β protein levels in the kidney tissues of HN rats (n = 6). (C) Rat body weights over 3 consecutive weeks after the formal experiment began, measured in each rat group (n = 6). (D) Levels of serum AST and ALT measured at week 3 following the initiation of the experimental protocol in each rat group (n = 6). Data are presented as means ± SEM. Statistical significance was determined using one-way ANOVA followed by Tukey’s multiple comparisons test (for A, B, and D) and two-way ANOVA followed by Bonferroni’s multiple comparisons test (for C). ^***^*P* < 0.001 versus the control group. ns, not significant versus the HN group. AST, aspartate aminotransferase; ALT, alanine aminotransferase.

**Figure S3. Analysis of pyroptosis and inflammation in HN.** (A) Gene expression of GSDMD in kidneys from mice of the HN group compared to the control group (GEO: GSE190205 and GSE262687) (n = 3). (B) Gene expression of *GSDMD* in renal tissues from control and HN rats based on RNA-seq data from our study (n = 3). (C) Levels of IL-18 and IL-1β secretion in UA-induced HK-2 cell supernatants measured by ELISA analysis. n = 3 independent experiments. Data are presented as means ± SEM. Statistical significance was determined using an unpaired Student's t-test (for A and B) and one-way ANOVA followed by Tukey’s multiple comparisons test (for C). A formal test for linear trend was confirmed using simple linear regression across the UA concentration gradient in (C) (*P_trend_* < 0.001). ns, not significant; ^*^*P* < 0.05, ^**^*P* < 0.01, and ^***^*P* < 0.001 versus the control group.

**Figure S4. The effects of *p*-CA on fibrotic markers in UA-induced human primary PTECs.** (A) Western blotting shows Collagen I, Fibronectin, and α-SMA protein, and protein level normalized to GAPDH in human primary PTECs. (B) Representative immunofluorescence images of Collagen I and Collagen III (Scale bar = 5 μm) and quantification of Collagen I and Collagen III fluorescence intensity in human primary PTECs. For A and B, n = 3 independent experiments. Data are presented as means ± SEM. Statistical significance was determined using one-way ANOVA followed by Tukey’s multiple comparisons test. A formal test for linear trend was confirmed using simple linear regression across the concentration gradients in (A) (*P_trend_* < 0.001). ^***^*P* < 0.001 versus the control group; ^###^*P* < 0.001 compared with the UA group.

**Figure S5. Analysis of the differentially expressed genes in HN mice from the GEO database.** (A) Volcano plots depicting DEGs in kidneys from HN mice compared to the control group (GEO: GSE262687). (B) Volcano plots depicting DEGs in kidneys from HN mice compared to the control group (GEO: GSE190205). Differentially expressed genes were identified using the Limma R package with a false discovery rate (FDR) < 0.05.

**Figure S6. Expression profiles of CTSB, evaluation of target selectivity, and validation of mutant stability.** (A) Western blotting and quantification of CTSB protein levels normalized to GAPDH in human primary PTECs. (B) Quantitative real-time polymerase chain reaction (qRT-PCR) analysis of *CTSB* mRNA levels in each HK-2 cell group. (C) Representative images of sequential section immunohistochemical staining for CTSB and aquaporin 1 (AQP1) in kidneys from HN rats. Scale bar = 50 μm. (D-E) Uniform Manifold Approximation and Projection (UMAP) analyses depicting distinct renal cell clusters, CTSB-positive cell localization, in CKD patient group (data from KPMP website). Black outlines indicate the PT cluster. (F) Dose-response curves evaluating the *in vitro* target selectivity of *p*-CA against recombinant human CTSB, CTSL, and CTSS using cell-free fluorometric screening assays. (G) Western blot analysis evaluating the basal expression levels of WT and mutant CTSB proteins in HK-2 cell lysates at 37°C prior to thermal challenge (Input controls for CETSA). For A, B, F, and G, n = 3 independent experiments. Data are presented as means ± SEM. Statistical significance was determined using one-way ANOVA followed by Tukey’s multiple comparisons test (for A, B, and G). For F, IC50 values were calculated by nonlinear regression of dose-response curves. ^*^*P* < 0.05 and ^***^*P* < 0.001 versus the control group or the Vector group. ns, not significant compared with the UA group or the WT group. TAL, Thick Ascending Limb; DTC, Distal Tubule Cell; PTC, Proximal Tubule Cell; LOH, Loop of Henle; SMC, Smooth Muscle Cell.

**Figure S7. Evaluation of lysosomal membrane permeabilization, NLRP3 and WWP1 mRNA transcription, and K63-linked ubiquitination.** (A) Evaluation of lysosomal membrane permeabilization (LMP) using Acridine Orange (AO) staining in HK-2 cells treated with UA (800 μM) alone or cotreated with *p*-CA (200 μM) for 48 h. The histogram shows the ratio of red (intact lysosomes) to green (cytosolic leakage) fluorescence intensity. Scale bar = 15 μm. (B) Western blot analysis of GAPDH (cytosolic marker) and LAMP1 (lysosomal marker) serving as reciprocal purity controls for the subcellular fractionation assay presented in main Figure 7B in HK-2 cells. (C) Quantitative real-time polymerase chain reaction (qRT-PCR) analysis of *NLRP3* mRNA levels across the indicated groups, showing no transcriptional suppression by CTSB inhibition. (D) Densitometric quantification of K63-linked ubiquitination of NLRP3 (from the Co-IP assay in main Figure 7E). The density of the K63-ubiquitin smear was normalized to the immunoprecipitated NLRP3 protein. (E) qRT-PCR analysis of *WWP1* mRNA levels in the indicated groups. For A, C, D, and E, n = 3 independent experiments. Data are presented as means ± SEM. Statistical significance was determined using one-way ANOVA followed by Tukey’s multiple comparisons test. ns, not significant. ^***^*P* < 0.001 versus the control group.

**Figure S8.** **Comparative effects of *p*-CA versus CA-074Me on body weight and serum transaminases *in vivo*.** (A) Rat body weights over 3 consecutive weeks after the formal experiment began, measured in each rat group (n = 6). (B) Levels of serum AST and ALT measured at week 3 following the initiation of the experimental protocol in each rat group (n = 6). Data are presented as means ± SEM. Statistical significance was determined using two-way ANOVA followed by Bonferroni’s multiple comparisons test (for A) and one-way ANOVA followed by Tukey’s multiple comparisons test (for B). AST, aspartate aminotransferase; ALT, alanine aminotransferase. ns, not significant; ^*^*P* < 0.05, and ^**^*P* < 0.01 versus the control group; ^#^*P* < 0.05, and ^##^*P* < 0.01 compared with the HN group.

**Table S1** **Clinical characteristics of patients from the hyperuricemia-associated CKD cohort in this study**

| **Age** | **Gender** | **Scr(μmol/L)** | **BUN (mmol/L)** | **SUA(μmol/L)** |
| --- | --- | --- | --- | --- |
| 67 | Female | 232 | 13.7 | 305 |
| 47 | Male | 126 | 9.4 | 373 |
| 54 | Male | 84 | 5.8 | 451 |
| 61 | Female | 72 | 6.3 | 355 |
| 72 | Female | 190.5 | 11.79 | 246.8 |
| 74 | Male | 96 | 4.3 | 350 |
| 54 | Male | 92 | 6.1 | 343 |
| 65 | Male | 166 | 10.3 | 348 |
| 41 | Male | 73 | 5.8 | 396 |
| 68 | Male | 117 | 6.6 | 482 |
| 43 | Male | 122 | 8.1 | 281 |
| 33 | Male | 105 | 6.2 | 545 |
| 73 | Male | 109.2 | 10.17 | 307.9 |
| 64 | Male | 123 | 10.3 | 413 |
| 78 | Male | 120 | 8.5 | 373 |

**Table S2 Selected phenolic compounds**

| **Number** | **Name** | **Structure** |
| --- | --- | --- |
| 1 | Ferulic acid |  |
| 2 | Chlorogenic acid |  |
| 3 | *p*-Coumaric acid |  |
| 4 | Caffeic acid |  |
| 5 | Phloretin |  |
| 6 | Curcumin |  |
| 7 | Gallic acid |  |
| 8 | Fisetin |  |
| 9 | Ellagic acid |  |
| 10 | Resveratrol |  |
| 11 | Piceatannol |  |
| 12 | Gastrodin |  |
| 13 | Kaempferol |  |
| 14 | Rosmarinic acid |  |
| 15 | Proanthocyanidins |  |
| 16 | Pterostilbene |  |

**Table S3 Sequences of small interfering RNAs (siRNAs) used in this study**

| **Target gene** | **Sense sequence (5′→3′)** | **Antisense sequence (5′→3′)** |
| --- | --- | --- |
| CTSB (si-CTSB) | GGUCAACUAUGUCAACAAATT | UUUGUUGACAUAGUUGACCTT |
| WWP1 (si-WWP1) | GAAGUCAUCUGUAACUAAATT | UUUAGUUACAGAUGACUUCTT |
| TRIM31 (si-TRIM31) | GGACCACAAAUCCCAUAAUTT | AUUAUGGGAUUUGUGGUCCTT |
| MARCHF7 (si-MARCHF7) | GCACUUGGGAGUAAUUUGATT | UCAAAUUACUCCCAAGUGCTT |
| Negative control (NC) | UUCUCCGAACGUGUCACGUTT | ACGUGACACGUUCGGAGAATT |

**Table S4 Functional roles and structural mapping of key CTSB residues**

| **Manuscript Description** | **Residue Index in PDB: 8B4T** | **Functional Role** |
| --- | --- | --- |
| Cys26 | Cys26 | Catalytic dyad |
| His199 | His199 | Catalytic dyad |
| Asn219 | Asn219 | Catalytic triad |
| His110 | His110 | Occluding-loop residue |
| Gln23 | Gln23 | Active site pocket |
| Gly198 | Gly198 | Active site pocket |
| Trp221 | Trp221 | Hydrophobic interaction |

Note: All residue numbering used in this manuscript strictly corresponds to the mature human CTSB crystal structure, PDB ID: 8B4T

**Table S5 Potential bioavailable targets of *p*-CA predicted by TCMSP, SuperPRED, SwissTargetPrediction, PharmMapper, ChemBL and STITCH databases**

| **Database** | **Targets** | **Number** |
| --- | --- | --- |
| TCMSP | PTGS1 PTGS2 CTRB1 PLA2G2E TYR CCL16 | 6 |
| SuperPRED | ESR1 TTR APEX1 TRIM24 ESR2 HSD17B10 KLF5 PIN1 NFE2L2 SLC6A5 CHRM5 HDAC5 TOP2A STAT1 FCGRT RORB PSMB1 QRFPR TDP1 CHRNB4 PDGFRB NFKB1 CLK4 FPR2 CHRM4 CTSD NQO2 FPR1 HTR2C MAOA NR1I2 GLRA1 CDK5 AVPR1B PTPN2 SLC2A1 CSNK2B CHRM3 NTSR2 CYP3A4 ALOX12 NR4A1 GRK5 THRA LGALS3 BMP2K CXCR4 CHRNA1 ADAM10 GRIN1 PTGS1 MARK4 GBA2 GUSB EPHB2 ACACB TNIK CDC25B CHRM2 CHRM1 ERAP1 CYP2A6 S1PR5 BLM CTSS PIK3R1 ACVR1B GSTP1 AAK1 NR3C2 STING1 NTRK3 KDM1A EPHB3 KDM6B TLR8 ULK3 ADORA3 METAP2 FGR PRCP MAP2K2 ITGB1 PSMB2 HDAC8 PDGFRA PLK4 TBXA2R | 88 |
| SwissTargetPrediction | AKR1B1 CA2 CA7 ESR2 CA1 CA3 CA6 CA12 CA14 CA9 CA4 CA5B CA5A MIF ALOX5 MMP9 MMP1 MMP2 PTPN1 AKR1B10 TLR4 CA13 KDM4E KDM4A KDM3A KDM6B FTO KDM4C F3 HSD11B1 HCAR2 TPMT DAO TRPA1 ESR1 PGR FYN LCK NGFR | 39 |
| PharmMapper | PIM1 CHEK1 GSTP1 KDR ESR1 PDE4B PDPK1 CSNK1G2 CDK2 CA2 PLK1 MTAP DCK ESRRG KIF5B MIF EPHB4 PRKACA CDK6 YARS1 SOD2 CDA AMD1 PDE4D LDHB PAH HSP90AA1 ME2 AKR1C3 AHCY ADH5 UCK2 LCK GALE AKR1B1 F10 SSE1 F2 TYMS MAPKAPK2 CAT FABP4 GSK3B MMP13 SRC PPARA AURKA THRA PPARG MMP8 HSPA1A HSPA1B FGFR1 DTYMK CCNA2 MAPK14 PTK2 TPH1 HCK RAF1 PTPN1 MMP9 ZAP70 ARG2 BST1 PAPSS1 APRT PDE5A FKBP1A ABL1 CTSS NNT ADK ARHGAP1 SULT1E1 DHFR ANG MMP12 CDC42 CTSF ACP3 PCK1 GPI HINT1 KIF11 RAN FDPS BCAT2 TEK MMP16 HEXB AR SULT2B1 TK1 GSTA1 DDX39B CASP3 PKLR RARG MAPK10 NT5M SETD7 F11 CTSB ITPKA NMNAT3 NR3C1 HADH RAB9A BACE1 RNASE2 OTC ABO MAP2K1 MAPK1 GSR HPRT1 KAT2B EIF4E ARF4 ATIC GSTA3 AMY2A DUT GSTM2 ALB WARS1 GART HMGCR GSTM1 GMPR RND3 Pfkfb1 RAB11A NME2 CLEC4M SELE F7 RAP2A RAC1 G6PD REN TGM3 BIRC7 RFK ESR2 INSR DCPS CMA1 MMP1 Arl5b GMPR2 TAP1 AK1 HNMT FGG CASP1 HRAS DAPK1 SULT2A1 GSTT2B APAF1 PPP1CC PNMT TPSB2 ELANE TRDMT1 MAPK12 RAB5A SULT1A1 UAP1 MME AKT1 MAOA HSD17B1 SPR AOFA_HUMAN SIRT5_HUMAN P14061 P35270 | 180 |
| ChemBL | DHFR F10 USP7 VCP IL2 GRM3 CASP3 CHRM3 SLC18A2 HRH1 PREP KDM4A CALCRL CASP1 KRAS KDM4B HRH2 PGR ACHE DHFR TBXAS1 PLK4 CHRM4 NPY2R PTK2 CASP8 PSMB9 ABCC1 ACHE PPARG CYSLTR1 EPHX1 LIMK2 ACHE ADRA2B ADRA1D IMPDH1 ADK PRMT3 F7 BRD4 ACHE SLC1A2 MC5R MGLL NR3C1 NOS2 REN BCL2 CHRM3 TRAP1 HDAC1 ADRA1A APLNR TDO2 LGALS3 KDM5C ITGA4 F10 CHRM5 CHIA MC3R ADRB3 METAP2 ADRA1D DOT1L PRMT5 ACACA MAPKAPK2 HSP90AA1 DHFR SLC5A2 AR ESR2 ADA NLRP3 ALPL CTSK FYN ACHE TMPRSS6 QPCT CHRM2 BCHE SLC9A1 DHODH HRH3 LCK HDAC6 ACE DHFR F11 CYP26A1 CFD GPR84 MC4R KLKB1 F9 IMPDH2 FGFR1 ADRA2A MGLL MALT1 WDR5 TDP2 CHRM1 CTSB PLAU AVPR1A ADRA2C CCR4 COMT ADRA1B BRAF BRD1 DHFR CCR5 ROS1 ATAD2 HDAC8 MCL1 TMIGD3 DHODH BACE1 CYP2A6 ADRB1 GRM2 DDR2 SPHK2 DHFR NOX4 EED PIN1 LDHA ADRB2 KDM5A FFAR2 KEAP1 GPBAR1 CCR2 LDHB CHRM2 CYP1A2 PLK1 NPY1R P2RX4 KAT6A PLG SLC5A1 HDAC3 KCNN3 YES1 GPR35 DHFR ATM | 155 |
| STITCH database | PTGR1 TYR LPO HAL CYP1A1 CYP1A2 EPX MPO TPO SULT1A1 | 10 |

**Table S6 Genes associated with HN summarized in DisGeNET, DrugBank, GeneCards, PharmGKB, TTD and OMIM database**

| **Database** | **Targets** | **Number** |
| --- | --- | --- |
| DisGeNET | HPRT1 XDH SLC2A9 ABCG2 APOE TGFB1 SLC5A8 ALDH16A1 ALB TNF HBA1 APLN CRP G6PC1 HBA2 HBB UMOD INS GPT SLC22A12 NLRP3 AGT LCN2 IL1B HAVCR1 SLC2A6 PPARG CXCL8 IL6 IL18 | 30 |
| DrugBank | XDH SLC22A7 AOX1 SLC22A2 SLC22A12 SLC22A11 CYP2C9 CYP3A4 SLC22A1 SLC16A7 ABCC1 SLCO1A2 SLC16A1 SLC22A6 SLC22A10 ABCC11 SLCO1C1 PANX1 CYP2C8 SLC2A9 TAS2R16 FABP2 CYP2B6 NR1I2 ABCB11 | 25 |
| GeneCards | UMOD HNF1B NLRP3 REN ALB HNFJ3 TGFB1 IL6 HPRT1 CCL2 INS MEFV MIR192 IL1B DARS2 TNF APOA1 IL18 SEC61A1 IL1RN APRT PKHD1 CST3 CRP APOE LOC107548112 CXCL8 MALAT1 TLR4 MYH9 SERPINE1 NPHP4 XDH PPARG NPHP1 HP SLC2A9 MMP9 GLA IL1R1 MUC1 NPHP3 LDHD LOC107032760 LOC129929047 SLC12A3 SLC22A12 PTGS2 HLA-B SLC12A1 TLR2 SOD2-OT1 HMOX1 PAX2 SLC22A11 S100A9 PRPS1 SLC17A1 TMX2-CTNND1 NOS2 CERNA3 SLC22A6 G6PC1 IL1RAPL2 LPL ABCG2 SIRT1 P2RX7 SLC17A3 PDZK1 LINC01672 NOD2 VDR PLAU RELA TNFSF11 IFIH1 MYD88 PKD2 GAS5 IL17A RIGI MIR155 PNP LEP MIR221 CASP1 NLRP1 DNAJB11 MIR146B FXYD2 RPL36A-HNRNPH2 MMP2 SNHG16 SLC22A13 MAPK1 PYCARD S100A8 R3HDM2 B3GNT4 TMEM171 TIMP1 ALPK1 SLC22A8 ADSL MIR223 PIK3CG IL6R KCNJ1 STAT3 LPA NFE2L2 F2 SYK CYP2E1 MIR488 CARMIL1 SLC37A4 GCG ALDH2 CD14 MMP3 CR1 HPN AFG2B CST9L TREM1 MIR146A CTSB MTNR1B PDCD1 SNORD15A CLCNKB LINC02605 HOTAIR CYP2C8 SLC22A7 SLC16A9 HOTTIP IGF1R MC3R SNHG5 MAPK14 SRC CSF1 MIR203A SNHG14 TRPV4 PTX3 CALCA MIR30C1 PRPS1L1 MIR663A MIR20B GYS1 UMPS MIR451A PRDM1 MIR561 SLC4A1 BMP2 MIR494 SNHG7 CCL20 MIR22 CLEC12A SLC17A5 MIR486-2 BRD4 CD44 INPP5D MIR185 STING1 HIPK3 AMPD1 ATIC ALDH16A1 KCNN4 G6PC2 SMAD5-AS1 CSF2 BDNF-AS IRAK4 ADA LRP6 SNHG8 TBX21 SNHG12 CARD8 PYGM HDAC6 FAH PRPSAP1 GAA POMC GAD1 EPHA2 GBE1 TEC ANK1 TH DGUOK SGCE PTGS1 AMPD3 NEK3 ATM SPTB NGF ADK MAP3K7 METTL6 PLA2G4A TERT NR2C2 LOC126862549 AGTR1 HNF1A AKR1B1 HFE IL10 LCN2 HNF4A KCNJ11 GCK NEUROD1 HAVCR1 ABCC8 PDX1 CD40LG PAX4 PTF1A BLK APPL1 KLF11 GAPDH IFNG WFS1 SEC63 PCBD1 GLIS3 RFX6 UBA5 UFM1 LHX1 UFC1 DDRGK1 DDX52 CDK5RAP3 KIF12 UFL1 ZNHIT3 SYNRG GGNBP2 C17orf78 ABCG5 EGFR RUVBL1 GNB3 LEPQTL1 MIR33B ADRB3 PTH HNF4G KIF3A CYP2C19 USP11 DDC COL4A3 ACE COL4A4 MTHFR ZNF365 SLC3A1 FASN FABP2 FABP4 SLC7A9 LOC126860945 CPT2 APOB TP53 B2M CBS GGT1 FABP3 NOS3 CD36 ADIPOQ ACP5 PPARA ALAD DAO HADHA ALOX5 FABP12 PIK3CA CPT1A CAT SOD1 PON1 ACP1 TTR VEGFA GBA1 MME CETP LIPC IGF1 AGT COL4A5 SOD2 GPT ACADS MAFB CLTRN H19 CD79A NAGLU EPO MTOR CASP3 FFAR2 NPHS1 MPO CYP3A4 ICAM1 G6PD EGF AKT1 FN1 MIR21 NEU1 HMGCL TAT FAS MYC CYP2C9 INSR SLC2A1 SPP1 AMBP AGXT MMUT HLA-DRB1 TNFRSF11B RBP4 CTNNB1 APOC3 ESR1 HMGCR TF CYP1A1 SIGLEC1 EDN1 JUN BCL2 SELE APP CFTR HBB GATM MTRR PVT1 CD4 IL2 BGLAP ABCA1 NPPB VWF IGFBP3 NAGS AR LRP2 CP LCAT CCK GLUD1 CDH1 UCP2 GRHPR ETFA PKD1 CYCS GHRL SLC2A2 TFRC ABCC2 ETFB GSR GSTP1 BMP6 CYP1A2 CASP8 CEL MB C3 VCAM1 HIF1A ABCB1 SERPINA1 SUOX CYP2D6 MAPK8 ACE2 COX5A IGF2 HGF F5 GAST TNFRSF1A SNCA CASP9 CFH AGER IL4 IFNA1 PPARGC1A RARRES2 NR1I2 BTD KCNQ1 JAK2 MAPT CDKN2A FGA OXA1L SLC34A1 PARP1 CDKN1A SGK1 RHOA HLA-DQB1 BAX MAT1A IRS1 THBD IL1A COMT APOC2 SCT NPPA GCKR HSPG2 FOS SREBF1 ARG1 BDNF SELP PLAT NPHS2 SST SERPINC1 NR3C2 TPO CCND1 GCH1 CDKN3 CA2 HMGB1 AVP RETN MOCS2 CTNS MMP1 MOCS1 ADRB2 MT-ND5 NR3C1 MT-TL1 SLCO1A2 MAPK10 PTEN DNAH8 CS C4A GSTM1 RYR1 FGF23 IDUA KNG1 UPB1 RAF1 TRPV1 THBS1 STAT1 GH1 FGFR2 ENPP1 APOL1 HLA-DQA1 APOA4 PCNA TYMS SELLCLU ALPP GUSB NFKB1 TG PGR-AS1 APOA5 TNFSF10 MIF PTPN11 ITGB3 JAK1 GHR CDK1 MT-TF SLPI CXCR4 AFP CCL3 BRAF PCSK9 FBN1 CYP2A6 PNLIP MT-ND2 F3 ANXA5 CNR1 PRNP ATP6V1B1 TSC1 CPOX ANPEP RAC1 FASLG TGFB2 HBA1 IL2RA PHGDH CSF3 LMNA BCL2L1 HSPD1 MT-ND1 IDO1 FGFR1 ENO2 GSTT1 KL CDK4 FOXP3 ADM SHC1 SOAT1 SLC9A1 MLXIPL IL13 PF4 MT-CO1 BMP4 PTK2 NQO1 SLC41A1 SLC22A2 GPX3 SOD3 AOX1 ALOX5AP TET2 SAA1 CNDP1 PLAUR SCARB1 NDUFS4 ITGAM CYP11B2 CDK2 TRPC6 HSPA4 ELANE S100B PRKAG2 PRKCD GNAS DCN EIF2S1 CD40 NFKBIA POLR1C TXN MCL1 BCHE HSD11B2 ELN NR1H3 IFNA2 MT-ND4 GUCY2D HSPA8 PIK3C2A IGFBP1 SLC13A2 AQP2 ADD1 TKT PGR GP1BA CD38 OCRL ITGA2 EMSLR MDM2 BIRC5 KLK3 LOC107133510 RB1 SLC5A6 RPS6KB1 IFNB1 PRKACA HSPA5 MIR217 MIR199A1 NR5A1 CGA MYCN SLC26A1 APOC1 MPL SMAD3 KDR RNASE3 TFAM TGIF1 PTPN6 NR5A2 FGF21 BCS1L MT-ND6 LTF TRA-TGC7-1 IL3 PCSK1 SERPINF1 IL15 EDNRA PLA2G7 MT-CO2 NEAT1 RELN CD59 CEP290 TNFRSF1B XIST PDGFRB KCNK5 PTGDS MT-CO3 TYMP ITGB2 CLCN5 ATF2 CYP27B1 KITLG NAMPT PRL LTA MIR125A ESR2 PRDX1 TIMP3 NAT2 LOC106099062 BDKRB2 FIBP APOH CHUK PRKD1 PAPPA CXCL12 CCR1 SAA4 TCF7L2 SLC9A3 ACAN LGALS1 NDUFAF6 CCNA2 CTSG PCSK2 CD55 PLD1 SLC19A2 SLC15A2 RUNX1 LYN MIR142 CTLA4 TSC2 PSPH SLC4A4 ITIH4 ATP7B ITPR1 FBXL4 LAP3 LEPR ALK ADH1B CDH2 CUBN GNRH1 CD9 CKB FYN MIRLET7C SHBG HCFC1 FOXO1 CXCL10 DLEU2 HLA-G MT-ND3 HOGA1 STAT5B NTRK1 PKLR CYBA PRKCSH MT-TP ENG RRM2B ADCY10 FGB FLT3 FTO LGALS3 GC PTK2B GANAB COG2 MYH7 SURF1 STAT5A CLDN16 SLC5A2 LCK TNFRSF11A HTR2A FLT1 SAFB CALR PROS1 CTSK MMP7 CFLAR MAF NTS CKM MMP8 IL11 MIR150 ANXA1 ST3GAL4 MIR27A ANGPT2 CCL4 KHK TNNT2 MMP14 IL2RB NDUFS1 MEN1 OSM MIR122 SYT1 PPIG AQP1 KEAP1 TNFRSF10B HLA-C LRP5 NADK2 CYP11A1 PKD2L1 LRP1 ALLC EDNRB IGFBP2 AHSG HAS1 ERBB4 SCARNA5 ALPL THPO MIR29C TNFSF13B F7 LCN1 MT-TS1 TLR9 MC4R EZH2 NOX4 AKAP13 XIAP ANGPTL4 PODXL ITGA4 MCOLN1 ALG5 ELAVL1 OLR1 PTPN22 TTC21B GATA3 ITGAL CA4 HNF1A-AS1 DVL2 SLC2A5 WNK1 MIR193A ADAM17 MASP1 HMGA2 CDK7 COQ9 NT5C3A XBP1 S100A4 HLA-DPB1 GLP1R IL18R1 PTPRC TNFRSF10A JAG1 SLC5A1 MIR17 GDF15 MICA KAT5 VHL PAX5 CKMT1B RPTOR IFI27 CYP24A1 TNC IL7 ALMS1 NDUFV1 CFP CDKN2B SPG21 PAFAH1B1 DUSP1 FOSL1 PHEX CCND3 MEP1B AOC3 POU5F1 VPS33B IL6ST CD46 TINAG FTH1 MT-TW REG1A BCR AVPR2 ASXL1 TRC-GCA24-1 H6PD TMEM126B DRD1 AIRE MT-TN DNM2 TACR1 CAPN10 FLNA CXCL9 DPEP1 CCND2 IFT140 SOCS3 SIRT6 SETD2 CKMT2 MYL2 WARS2 BDKRB1 LINC01554 BCL2L11 CXCR3 MMP12 TRPC1 SYVN1 MIR23B HSD3B1 MT-TS2 MT-TH DNTT HES1 RAG1 MAP3K1 C5 ZAP70 ATP6V0A4 KCNJ5 COL3A1 NFAT5 TAOK1 PRKACG BAK1 PAX8 HAMP FCER2 SNORD118 EIF4A1 MIR145 BSND NHERF1 CDKN2B-AS1 POLI MT-TQ PGA5 NDUFS2 ESRRA APLN IGF2BP2 MIR143 NDUFS3 C1GALT1C1 ANGPTL3 SKI CALB1 NDUFAF1 LOC130059760 B4GALT1 IL12B PGA4 NOX1 BCL6 NOD1 AQP3 CCR2 SERPINH1 HLA-E MPC1 AZGP1 SRSF6 PGA3 CABIN1 PSMD9 SLC4A8 MUC16 FIS1 JAK3 YY1 REL PGF HNRNPU SERPINF2 NT5E SCO2 INVS SIK2 P2RX4 PFKP ITGAV MOK NDUFV2 CD19 CLDN19 WNT9B PKD1L3 TYK2 NDUFS8 ATP6AP2 DHFR2 UTS2 PIGN TXNIP SLC22A3 SLX1A-SULT1A3 NOTCH2 SLC34A3 TIMMDC1 SORT1 MIR106B BIRC3 LGALS9 AHSP BMPR2 TNFRSF8 PSTPIP1 NIPSNAP2 BIRC2 CDKN1C DEFB1 RNU4ATAC PSMB9 MIR9-1 CFL1 ADNP NFATC1 USP2 BAMBI SESN2 ZMIZ1 PXDN DCDC2 CLCNKA MIR93 RPGRIP1L DICER1 MIRLET7B SHROOM3 MIR485 VIPAS39 BBS1 HSP90B1 MT-RNR2 LOC126859690 CXCL5 IL12A SPI1 ESRRG S100A6 SLC26A6 PPM1A C12orf43 STAT4 RIPK1 ALPI STXBP2 H3C1 TAGLN MCAM NHERF2 MIR92A1 PLSCR3 IL1RAP CR2 TMEM67 TNNI3 TBXT CXCR5 STC1 CPB2 RNASEH2C MIR320A FCGRT KCNE1 BTNL2 POSTN CTCF COQ5 MPDU1 ACVR1 TRPV5 HIPK2 HTRA1 IL2RG AIF1 FCGR2B TNFSF12 KMT2D COL18A1 LOC126805749 MBD2 HYOU1 UHRF1 TRAF1 SSTR3 CXCL16 PGC ACVRL1 SAA2 NLRC4 NFATC3 NDUFS7 C11orf65 PRKACB RAP1B TENM2 PPM1D SFN DZIP1L SH2D1A ADGRL2 PAAF1 NSD1 A2M INS-IGF2 EPHB4 RBMS1 RMND1 NEK8 ADAMTS5 STX16 IRF5 TULP3 KLF15 GNAI3 ENTPD5 RCAN1 PSRC1 FMN1 THY1 CNKSR3 RPS15 CASQ1 NEK7 ORAI1 OFD1 ANKS6 RREB1 C4BPB IFT172 MSTO1 DUSP9 LPP DACH1 IFT88 INSRR MIR222 ALG6 CLDN10 NEK9 MMP10 GLI2 CELSR2 BAZ1B TBL2 GRB10 DYNC2H1 IL10RA ARL13B PARP11 BAIAP2 WWTR1 CDKAL1 MUS81 PSORS1C1 CD27 NUP107 FAM20A MIR197 AAAS TRIP10 MKS1 ARL2 MIR1207 RAPSN AICDA MIRLET7D CFTR-AS1 CD163 ARID4B CCR6 TCF4 TGFBR3 BBS4 PRDM8 MIR127 LOC102723407 GPHA2 NUDC SH2B3 MS4A1 CD24 CCL15-CCL14 SERPINA12 EPB41L3 CXCL13 PKDREJ DOCK8 SLC14A1 KLF10 BRCC3 TIGAR MIR30B GALNT3 IPPK ITM2B USP25 PRRC2A MIR211 PKD1L1 TNFRSF13B SBF2 FAT4 BMI1 MMP16 IFT81 WNT7A SPPL3 CISD1 AK3 MLLT3 ROCR AQP11 MCTP2 PKD1P6 ANKRD55 ADGRV1 UIMC1 USP34 ITPK1 CREB5 C1QTNF5 STX8 B4GALT1-AS1 PKD1P3 GREM2 TCL1A MFSD12 WWC1 ZDHHC24 LARP4B IFT20 SLC22A18 PKD2L2 VPS37D SOX11 KIAA0753 MIR10B SPINK4 IQCB1 MIR497 CYS1 CMIP CCT3 TRMT112 AIM2 AXDND1 SPRR2F CD248 OIT3 MIR181D NDE1 MICALL2 TMEM11 FCRL3 KIAA0319 TNS1 SOX15 POU3F4 PAX8-AS1 MIR499A PKD3 PKD1P1 EXOC5 MIR34B INTU TCF21 BCAS1 NAIP PKD1P2 PKD1P4 PKD1P5 TNFSF12-TNFSF13 PKD1P6-NPIPP1 MIR296 MIR324 ENSA TMEM187 LOC110283621 MYEOV MBL1P IFT52 R3HDML-AS1 NTN4 CNTROB IL22RA1 TET2-AS1 CD68 KCNQ1-AS1 ESM1 MIR587 LINC01362 MIR26A2 DZIP1 DYDC1 GLMP CLEC16A CCDC68 BOD1 CLEC4F PDILT DYDC2 RF00017-7825 TRIM65 MED20 CLDN14-AS1 IRX3 TENM2-AS1 LNCTSI MIR23C MIR665 | 1299 |
| PharmGKB | PRG4 NPHP4 NPHP3 GJB1 PNPLA6 NPHP1 PAX8 OSR1 CFH CEP290 FGF8 BMP7 ARMS2 WT1 C4orf54 KLHL40 KBTBD13 INVS IRX1 NPHS1 SDCCAG8 CMYA5 NPHS2 IQCB1 ETHE1 CCN3 TPM2 REN CFL2 NEB MLC1 SGCG HOXC11 CLDN16 NPNT GLIS2 TNNT1 SIX2 AIRE TSHZ3 GDNF GDF11 HOXD11 AVPR2 SLC5A1 HOXA11 ACTA1 SPRY1 ITGA8 ID3 RDH10 ROBO2 EYA1 FGF10 PAX2 BMP4 PLCE1 MYH6 NIPBL FOXC2 ZBTB16 CITED1 NF1 SLIT2 GLI3 BCL2 SHH | 67 |
| TTD | SLC5A4 AKR1B1 PTGIR URA ABCC1 ANO XDH PPARG PPARA URAT1 FABP1 CXCR2 CXCR1 COX-2 COX-1 GLUT9 IL1B NLRP3 | 18 |
| OMIM | MIG6 C1QA C1QC C1QB FCN3 SMPDL3B MMACHC PRDX1 PODN JAK1 UOX VUR1 PTPN22 RFH1 IL6R MUC1 PPOX SELL SELE PDCN ARPC5 PTGS2 CFH CFHR3 CFHR1 CFHR5 GFND1 REN SLC41A1 PIGR FCAMR MIA3 AGT NLRP3 MPV17 HNFJ3 PLEKHH2 LGALSL PPP3R1 ALMS1 NPHP1 IL1A IL1B IL1RN PROC ZEB2 PLA2R1 FN1 SMARCAL1 IGAN2 COL4A4 COL4A3 PPARG CCR5 TREX1 COL7A1 ROBO2 SEC61A1 IFT122 NPHP3 RARRES1 B3GALT3 SLC2A2 MUC20 SLC2A9 WDR19 SLC30A9 SCARB2 COQ2 ABCG2 IDDM23 FGA FAT1 POC5 IL13 CD14 GFPT2 MVCD7 HFE IDDM1 MBNP NEU1 CFB HLA-DQA1 ITPR3 VEGF CLIC5 MMUT RHAG PDSS2 IGAN1 CCN2 SLC2A12 SGK1 SOD2 THSD7A IL6 ELMO1 PGAM2 PON1 PON2 EPO PAX4 AKR1B1 SSBP1 ASAH1 SFRP1 UNC13B HNP1 INVS GSN LMX1B COL5A1 NOTCH1 AGPAT2 PTGDS GATA3 ZEB1 SGPL1 OIT3 PAX2 CD151 HBB DEL11p13 WT1 F2 SCGB1A1 MIR192 CCND1 APOA1 SLC37A4 HMBS NEDE TMEM218 LTBR IPO8 PFKM AQP2 NUP107 LYZ HNF1A BRCA2 ALG5 SPRY2 CLDN10 LIG4 COL4A1 VIPAS39 BDKRB2 IGH A1 B2M FAH PLIN1 VPS33B IFT140 PKD1 MEFV GP2 UMOD CD19 LDHD APRT CTNS CCL13 CCL5 HNF1B KRT14 G6PC ACE TTR SMAD7 NEDD4L SERPINB7 CNDP1 ZNF236 LMNB2 TNFSF14 ACP5 TGFB1 PLAUR FCAR ZNF543 MGME1 SNHG17 T2D3 MAFB PRODH MYH9 XPNPEP3 A4GALT WAS GLA PRPS1 COL4A5 GPC3 HPRT1 AC ADTKD1 AGS1 AIIDE ALDR1 ALRH ALSS AMYLD2 AMYLD4 AMYLD5 AORF APP3 APRTD ARC16 ASML3B ATS1 ATS2 ATS3A BCRP BF BSVD1 C11DELp13 C1QD1 C1QD2 C1QG C20orf72 C4orf1 C5orf37 CALNB1 CD36L2 CED12 CELIAC1 CFHL5 CIAS1 CMKBR5 CMT1J CMTX5 CN1 COQ10D1 CTGF CVID3 DCP1 DFNB103 DLACD DLP1 DPD1 EBS1A ECYT6 EDSCL1 ELAM1 FANCD1 FAT FHR1 FHR3 FN FRP G6PT G6PT1 GFAT2 GLCT3 GLUT12 GLUT2 GLUT9 GSD7 HAKA1 HARP HDR HF1 HLA-H HPALP2 HPRT HVEML IFNB2 IGAN3 IL6RQ IMD2 IMD43 INV JBTS39 KDIDAR KIAA0439 KIAA0590 KIAA0960 KRML LIG4S LMN2 LPAAB LYAM1 LZP MADH7 MEF MEGSIN MHA MIRN192 MNSOD MODY9 MTDPS6 MUT MVCD1 MVCD2 MVCD4 NDI2 NDNC8 NEU NPH1 NPH3 NPHPL2 NPHS2 NPHS4 NPS1 NUP84 OSPL P1PK PALB PAPRS PBGD PC PEP PETA3 PGAMM PKD7 PLA2R PLIN PON PPARG1 PRAD1 PRODH2 PRXI PUM RALT RANBP8 RH50A RTD SAX3 SCYA13 SCYA5 SDYS SEC61 SERPINA8 SPENCDI SPL SRTD5 SSBP TAN1 TANGO1 TCF1 TCF2 TCF8 THPH1 TIG1 TNFCR UGB UNC13 URKR VIPAR VP WDR10 ZFHX1B ABCP ACE1 ADTKD2 ADTKD4 ADTKD5 AMYLD3 AMYLD6 AOS5 ATD5 ATS3B BCL1 BFH1 BHR1 BROVCA2 BSCL BSF2 C1QD3 C6orf210 CC10 CCCKR5 CDG2W CED CED1 CMT2EE CPETRL3 CRCS3 CRV D17S136E DFNX1 DIRA EBS1B ECYT5 EPM9 ESA FCU FHR5 FMDMF FMF FPLD4 FSGS10 FSGS7 FTNS GBG GLOB GSD10 HANAC HDRS HFE1 HFL1 HLF4 HNF2 HUEL HUS IL6QTL IMD113 KIAA0328 KIAA1568 KPD LAM1 LETS LIMPII MCM MCTO MODY3 MSA1 MTDPS11 MUNC13 MVCD6 NCC1 NIL2A 2-Nov NPHP2 NPHPL1 NPHS11 OHST OPA13 PAGA PFIC12 PPARG2 PPKN PTPN8 RENI RHPD1 RPRGL2 RSP5 SARP2 SCZD4 SFA1 SGBS1 SIAL1 SIOD SLSN1 SMADIP1 SMAPME SPE39 SRN1 SRTD9 TANGO THC1 THPH3 UAQTL2 UPS VISS VPCO WAGR AHUS4 AMRF AOVD1 ARMD4 BFH2 BILAPES BSCL1 C14orf133 CCSP CFHL1 CFHL3 CFHR5D CIMT1 COQ10D3 CRMO2 CVID15 DBAL DFN2 DFNA17 DURS3 EBS1C ENCEP FCAS1 FNZ GLM3 HELIX HERNS HIES5 HSF IBDIMDE ICH IDDM20 IDDM22 JBTS4 KIAA0268 LNHR LYP MCPH27 MER2 MHC1D4 MKS7 MVCD3 MVCD5 MZSDS NKEFA NPH2 NPHP13 NPHS14 O DG6 PAAND PPCD3 PVNH7 RCAD RHNR SCNX SIP1 TCP228 THC4 UAQTL1 AHUS1 ARMD14 BDPLT6 BSVD CED4 EBS1D EBS7 EPM4 FECD6 GFND2 GLM1 GOUT1 HGF LENCEP NALP3 ODCD2 PNCA2 RP80 RVCLS T2D ADTKD3 CFBD MATINS PYPAF1 RATOR SMDCF SPGF72 DFNA34 PADMAL KEFH AORF APP3 BSVD1 CFHL5 IGAN3 NPHPL2 PETA3 FHR5 HANAC NPHPL1 SFA1 CFHR5D ICH MER2 BSVD EBS7 RATOR PADMAL ACOT7 ACTRT2 AGMAT AGRN AGTRAP AJAP1 AKR7A2 AKR7A3 ALDH4A1 ALPL ANGPTL7 ARHGEF10L ARHGEF16 ARHGEF19 ATAD3A ATAD3B ATAD3C ATP13A2 AURKAIP1 B3GALT6 C1QTNF12 CA6 CALML6 CAMTA1 CAPZB CASZ1 CDK11A CDK11B CELA2A CELA2B CENPS CEP104 CFAP74 CHD5 CLCN6 CLCNKA CLCNKB CLSTN1 CORT CPTP CROCC CTNNBIP1 CTRC DDI2 DDOST DFFA DFFB DHRS3 DISP3 DNAJC11 DNAJC16 DRAXIN DVL1 ECE1 EFHD2 EIF4G3 EMC1 ENO1 EPHA2 ERRFI1 ESPN EXOSC10 FAAP20 FBLIM1 FBXO42 FBXO44 FBXO6 GABRD GNB1 GPR153 H6PD HES2 HES3 HES4 HES5 HP1BP3 HSPB7 HSPG2 HTR6 ICMT INTS11 ISG15 KAZN KCNAB2 KIF17 KIF1B KLHL17 KLHL21 LRRC38 LRRC47 LZIC MAD2L2 MASP2 MEGF6 MFAP2 MFN2 MIB2 MICOS10 MIIP MMEL1 MRTO4 MTHFR MTOR MUL1 MXRA8 NADK NBPF1 NECAP2 NMNAT1 NOC2L NOL9 NPHP4 NPPA OTUD3 PADI1 PADI2 PADI3 PADI4 PADI6 PANK4 PARK7 PAX7 PDPN PERM1 PEX10 PEX14 PGD PHF13 PIK3CD PINK1 PLA2G2A PLA2G2D PLA2G2E PLA2G2F PLA2G5 PLCH2 PLEKHG5 PLEKHM2 PLOD1 PRDM16 PRDM2 PRKCZ RBP7 RCC2 RER1 RERE RNF207 RPL22 RSC1A1 SAMD11 SCNN1D SDF4 SDHB SKI SLC25A33 SLC25A34 SLC2A5 SLC2A7 SLC35E2B SLC45A1 SLC66A1 SMIM1 SPEN SPSB1 SRM SSU72 SZRD1 TARDBP TAS1R1 TAS1R2 TAS1R3 THAP3 TMEM240 TNFRSF14 TNFRSF18 TNFRSF1B TNFRSF25 TNFRSF4 TNFRSF9 TP73 TPRG1L TRE-TTC3-1 TRG-CCC1-1 UBE2J2 UBE4B UBIAD1 UBR4 UBXN10 USP48 UTS2 VPS13D VWA1 WRAP73 ZBTB17 ZBTB48 | 761 |

**Table S7 Common target proteins selected for network pharmacology and RNA-seq**

| **Uniprot ID** | **Protein Description** | **Name** |
| --- | --- | --- |
| P07858 | Cathepsin B | CTSB |
| P03372 | Estrogen receptor | ESR1 |
| P17931 | Galectin-3 | LGALS3 |
| Q07869 | Peroxisome proliferator-activated receptor alpha | PPARA |
| P02768 | Albumin | ALB |
| P42574 | Caspase-3 | CASP3 |
| P08253 | 72 kDa type IV collagenase | MMP2 |
| P19838 | Nuclear factor NF-kappa-B p105 subunit | NFKB1 |
| P12821 | Angiotensin-converting enzyme | ACE |
| P09619 | Platelet-derived growth factor receptor beta | PDGFRB |
| P43235 | Cathepsin K | CTSK |
| P04798 | Cytochrome P450 1A1 | CYP1A1 |
| P08473 | Neprilysin | MME |
| O00206 | Toll-like receptor 4 | TLR4 |
| P04040 | Catalase | CAT |
| P30613 | Pyruvate kinase PKLR | PKLR |
| Q86WV6 | Stimulator of interferon genes protein | STING1 |
| P28065 | Proteasome subunit beta type-9 | PSMB9 |
| P14174 | Macrophage migration inhibitory factor | MIF |
| P06276 | Cholinesterase | BCHE |

**Table S8** **Binding free energies and energy components of CTSB-*p*-CA predicted by MM/GBSA (kcal/mol)**

| **System name** | **CTSB-*p*-CA** |
| --- | --- |
| Δ*E*_vdw_ | -11.78±2.36 |
| Δ*E*_elec_ | 81.41±6.24 |
| ΔG_GB_ | -82.50±3.25 |
| ΔG_SA_ | -2.50±0.27 |
| ΔG_bind_ | -15.38±1.83 |

ΔE_vdw_: van der Waals energy, ΔE_elec_: electrostatic energy, ΔG_GB_: electrostatic contribution to solvation, ΔG_SA_: non-polar contribution to solvation, ΔG_bind_: binding free energy.
